# Cave bacteria-induced amorphous calcium carbonate formation

**DOI:** 10.1038/s41598-020-65667-w

**Published:** 2020-05-26

**Authors:** Nóra Tünde Enyedi, Judit Makk, László Kótai, Bernadett Berényi, Szilvia Klébert, Zoltán Sebestyén, Zsombor Molnár, Andrea K. Borsodi, Szabolcs Leél-Őssy, Attila Demény, Péter Németh

**Affiliations:** 1 Department of Microbiology, Faculty of Science, Eötvös Loránd University, Pázmány P. sétány 1/C, H-1117 Budapest, Hungary; 2 Institute of Materials and Environmental Chemistry, Research Centre for Natural Sciences, Magyar tudósok körútja 2, H-1117 Budapest, Hungary; 3 Deuton-X Ltd., Selmeci u. 89, H-2030 Érd, Hungary; 4 Department of Earth and Environmental Sciences, University of Pannonia, Egyetem út 10, H-8200 Veszprém, Hungary; 5 Department of Physical and Applied Geology, Faculty of Science, Eötvös Loránd University, Pázmány P. sétány 1/C, H-1117 Budapest, Hungary; 6 Institute for Geological and Geochemical Research, Research Centre for Astronomy and Earth Sciences, Budaörsi út 45, H-1112 Budapest, Hungary

**Keywords:** Biogeochemistry, Environmental sciences, Materials science

## Abstract

Amorphous calcium carbonate (ACC) is a precursor of crystalline calcium carbonates that plays a key role in biomineralization and polymorph evolution. Here, we show that several bacterial strains isolated from a Hungarian cave produce ACC and their extracellular polymeric substance (EPS) shields ACC from crystallization. The findings demonstrate that bacteria-produced ACC forms in water-rich environment at room temperature and is stable for at least half year, which is in contrast to laboratory-produced ACC that needs to be stored in a desiccator and kept below 10 °C for avoiding crystallization. The ACC-shielding EPS consists of lipids, proteins, carbohydrates and nucleic acids. In particular, we identified large amount of long-chain fatty acid components. We suggest that ACC could be enclosed in a micella-like formula within the EPS that inhibits water infiltration. As the bacterial cells lyse, the covering protective layer disintegrates, water penetrates and the unprotected ACC grains crystallize to calcite. Our study indicates that bacteria are capable of producing ACC, and we estimate its quantity in comparison to calcite presumably varies up to 20% depending on the age of the colony. Since diverse bacterial communities colonize the surface of cave sediments in temperate zone, we presume that ACC is common in these caves and its occurrence is directly linked to bacterial activity and influences the geochemical signals recorded in speleothems.

## Introduction

Amorphous calcium carbonate (ACC) is known as a precursor phase of crystalline CaCO_3_ that plays a key role during calcium carbonate precipitation and biomineralization^1^. It is the least stable CaCO_3_ modification that rapidly transforms to crystalline calcium carbonate polymorphs. Laboratory-synthesized ACC crystallization can be delayed by keeping the physisorbed H_2_O below the critical level and storing the material in a desiccator and keeping it below 10 °C^2^. Additives such as Mg^2+^, phosphate, and organic macromolecules can retard its crystallization^3–5^. According to Purgstaller *et al*.^6^, the metastability of Mg-ACC is associated with the formation kinetics (pH and the Mg/Ca ratio) of the less soluble crystalline phase, i.e., the physico-chemical conditions of the environment.

Biogenic activity can also modify the physico-chemical conditions, and thus can enhance the preservation of ACC. It has been reported from tissues of various eukaryotic organisms and several organic molecules have been associated with its occurrence. According to Aizenberg *et al*.^7^, the skeletal parts of calcareous sponge *Clathrina* and the spicules of ascidian *Pyura pachydermatina* contain ACC and its formation is associated with polysaccharides and proteins enriched in glutamic acid (and/or glutamine), serine and glycine. ACC was also described from the intraskeletal organic matrix of numerous scleractinian corals^8^, the spicules of the embryos of *Strongylocentrotus purpuratus* sea urchins^9^ and the shell of *Biomphalaria glabrata* snail embryos^10^. Amines, glycosylated proteins and phosphoproteins were reported to preserve ACC in earthworm’s calciferous gland^11^ and the gastroliths of the red claw crayfish *Cherax quadricarinatus*^12^.

Bacterial cell surface shows favorable conditions for calcium carbonate precipitation provided by the cell wall and the extracellular polymeric substance (EPS). According to literature data, carboxyl, phosphate, hydroxyl and sulfate functional groups of the cell walls^13^ and EPS^14,15^ are polarized or getting deprotonated and become negatively charged, so that they can bind and accumulate cations (Ca^2+^, Mg^2+^, Fe^3+^, Cu^2+^, Na^+^, K^+^, Mn^2+^, Zn^2+^, Au^3+^ and Ni^2+^)^13,16^. Therefore, Ca^2+^ might locally reach high concentrations and as a result of the bacterial metabolism, the pH is increased favoring calcium carbonate precipitation^17–21^. During this process, ACC might precipitate first and transform to crystalline calcium carbonates. ACC formation was reported in the cultures of vaterite producing *Lysinibacillus* sp. GW-2^20^, Mg-calcite producing *Curvibacter lanceolatus* HJ-1^21^ and aragonite producing *Synechococcus leopoliensis* PCC 7942^18^. In natural environments, ACC nanoparticles embedded in microbial biofilm were detected in Eryuan, Gongxiaoshe, and Zhuyuan hot springs (China)^22^ and marine ooids from the Bahamas^23^. Although, microbial ACC precipitation is attributed to the cell surface, the details of its formation and transformation are unknown.

Microbial metabolism plays a significant role in calcium carbonate precipitation in aphotic subsurface environment as they locally modify the physico-chemical conditions. In particular, bacteria isolated from the surface of calcium carbonate caves induce calcite^17,19,24^, aragonite^24^, vaterite^19,24^, and monohydrocalcite^19^ formation in laboratory experiments. Since microbes colonize the surface of cave sediments (speleothems), they can significantly influence the type of precipitating calcium carbonates. Demény *et al*.^25^ reported ACC formation from the calcium carbonate precipitates of Baradla Cave (Hungary). The finding was rather surprising as the cave temperature was 11 °C, at which condition ACC is unstable^2–6^. However, the cave-originated ACC was not only present, but it did not crystallize. Although detailed microbiological investigations have not been conducted in Baradla Cave yet, it was expected that diverse bacterial communities colonize the surface of calcium carbonate speleothems. Thus, it is plausible that microbes can influence the preservation of ACC.

Here we show that numerous bacterial isolates from Baradla Cave induce ACC formation and their EPS shields it from crystallization for at least 26 weeks at room temperature. We studied the composition of the shielding material and suggest that ACC might be enclosed in a micella-like formula within the EPS that inhibits the water infiltration into it. Time-resistant ACC can provide the possibility of the structural characterization of this enigmatic material.

## Results and Discussion

### Characterization of bacterial strains

Dripping water samples were collected from Baradla Cave in Hungary as part of the evaluation of the microbial community colonizing the speleothems. The cultivation revealed the presence of a variety of heterotrophic bacteria with different colony morphologies on B4 agar medium (see Experimental section) and several strains were isolated from the medium to examine the effect of the microbial activity on calcium carbonate precipitation. Colonies of strains BaSD-214, BaSD-223 and BaTD-248 were shining and mucoid after 3 days cultivation at 21 °C indicating the presence of EPS (Supplementary Table S1). Hence these strains were selected to examine microbially induced calcium carbonate precipitation in detail. The 16S rRNA gene sequence of the strains BaSD-214, BaSD-223 and BaTD-248 showed 99.1–100% sequence similarity with the type strains of *Stenotrophomonas maltophilia, Bacillus simplex* and *Rhodococcus degradans*, respectively. These species have been detected from numerous environmental samples, in particular from soil^26–28^. The genera *Stenotrophomonas, Bacillus* and *Rhodococcus* have been commonly identified as part of the heterotrophic bacterial communities colonizing calcium carbonate cave speleothems^19,24,29^.

Bacterial cell surface molecules, as the cell wall and the encapsulating EPS have elemental role in the process of calcium carbonate precipitation, as these components bind and concentrate Ca^2+^ ions. The selected three bacterial strains possess comprehensively different cell wall structure. *Stenotrophomonas maltophilia* belongs to the Gram-stain negative bacteria and has an outer unit membrane containing high amount of lipopolysaccharides (LPS). Its LPS layer consists of an O-specific polysaccharide (3-O-methylxylose and l-rhamnose); a core oligosaccharide (D-glucose, D-mannose phosphate, D-galactosamine, D-galacturonic acid and a 3-deoxyoctulosonic acid) and a lipid-A component (phosphorylated glucosamine residues with N-fatty acyl and O-fatty acyl substituents)^30^. *Bacillus* has Gram-stain positive cell wall type with a dense murein (peptidoglycan) layer consisting of N-acetylglucosamine, N-glycolylmuramic acid, teichoic acids and lipoteichoic acids^27^. Endospores of *Bacillus simplex* are coated with an outer protein layer called exosporium^31^. *Rhodococcus* peptidoglycan is also Gram-stain positive and consists of D-and L-alanine, D-glutamic acid, meso-diaminopimelic acid as well as arabinose and galactose^32^. Mycolic acids (2-alkyl branched, 3-hydroxy long-chain fatty acids) consisting of approximately 32–38 carbon atoms are also present in the cell wall of *Rhodococcus degradans*^28^. Acid–alcohol-fast character of this cell wall is attributed to the mycolic acids.

To determine the biochemical properties of the bacterial strains, metabolic activity tests were applied. The tests (Supplementary Table S1), in consistent with previous reports^27,28,33^, confirmed that *Stenotrophomonas maltophilia* BaSD-214, *Bacillus simplex* BaSD-223 and *Rhodococcus degradans* BaTD-248 strains are chemoorganotrophic heterotrophic bacteria, i.e., they conserve energy from organic compounds and use them as their carbon sources. The strains were capable of the utilization of a broad range of carbohydrates (including glucose), proteins, amino acids and urea. Furthermore, we found that *Stenotrophomonas maltophilia* BaSD-214 could actively reduce nitrate to nitrite and ammonia as well.

In cave environment, organic and inorganic matters infiltrate through dripping water and concentrate on the surface of speleothems. Bacterial cells colonize this surface, proliferate and produce additional organic and inorganic matter, creating a biofilm structure. The detected biochemical activities confirmed that the studied bacterial strains are able to produce NH_3_ and HCO_3_^-^ through ureolysis and capable of dissimilatory nitrate-reduction and NH_3_ production from organic nitrogen compounds. The metabolic activities increase the pH in their microenvironment and result in CaCO_3_ precipitation. Thus, microbially induced calcium carbonate precipitation may actively contribute to the formation of cave speleothems.

### Calcium carbonate precipitation experiments

Calcite and vaterite precipitation were reported for strains of *Stenotrophomonas maltophilia* on solid B4 medium^34^ and *Bacillus simplex* in liquid urea-CaCl_2_ medium^35^. The calcium carbonate precipitating capacity of *Rhodococcus degradans* has not been tested before. We applied liquid and solid B4 medium to examine the effect of the bacterial activity on the crystallization process of calcium carbonate. These media are widely used for studying mineral precipitation. However, the study of calcium carbonate precipitating capability of the bacterial strains in liquid medium led to alarming results. TEM images showed abundant nanocrystalline vaterite even in the control (non-inoculated) sample (Supplementary Fig. S1). We attributed vaterite formation to CO_2_-degassing during the autoclaving procedure of the liquid medium preparation. In contrast, calcium carbonate precipitates were not observed in the control solid media, thus we applied them in our study. This finding questioned previous vaterite-precipitating bacteria results using liquid media^20^ and pointed to the applications of solid media for bacterial CaCO_3_ precipitations tests.

### Bacterial ACC precipitates in the colony incubated for 26 weeks

All three isolates could precipitate copious amounts of calcium carbonate (Supplementary Fig. S2). Visible precipitates of 200–500 µm diameter were observed along the edges and within the colonies after two weeks of incubation at 21 °C on solid medium. The size and amount of crystals increased with time (Supplementary Fig. S3). In case of *Stenotrophomonas maltophilia* BaSD-214 and *Rhodococcus degradans* BaTD-248 the whole colonies became calcified after 26 weeks of incubation by the time the agar plates completely desiccated. However, crystals appeared only in the central part of the colonies of *Bacillus simplex* BaSD-223. There were three strains that did not show calcium carbonate precipitating activity under same culture conditions, namely *Staphylococcus xylosus* BaTD-289, *Herbiconiux solani* BaOD-269 and *Agromyces subbeticus* BaOD-279 (Supplementary Fig. S4). These strains were taken as a negative control for carbonate precipitation.

**Figure 1:**
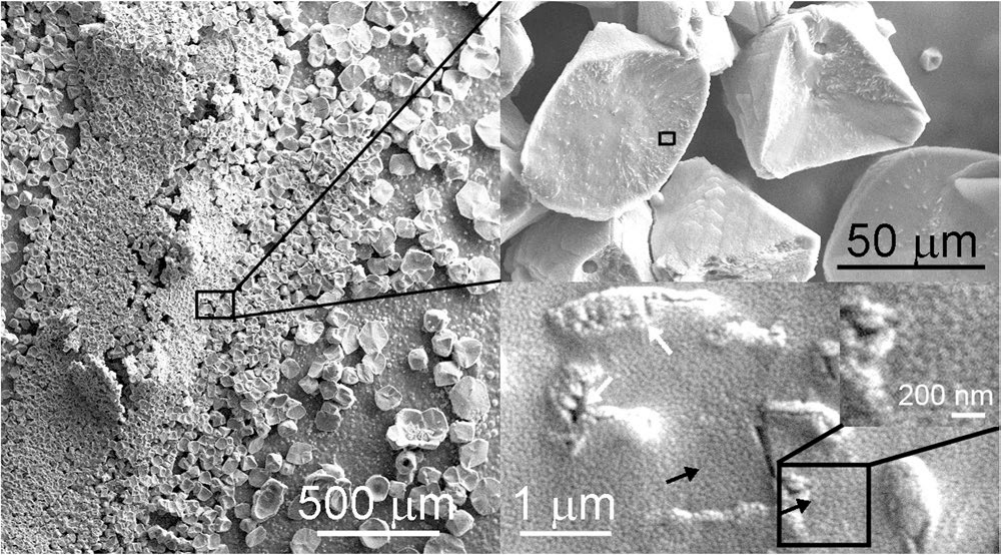
50-100 nm-sized calcium carbonate objects (black arrows) occur on bacterial residues and calcite surface precipitated by *Stenotrophomonas maltophilia* BaSD-214 incubated for 26 weeks. White arrows point to blown-up calcified bacterial molds deteriorated in vacuum.

**Figure 2:**
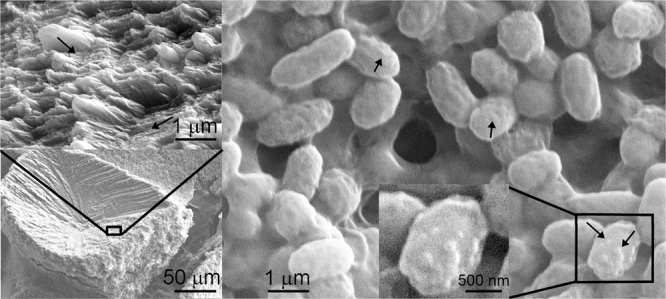
Nanosize calcium carbonate globules (black arrows) occur on precipitated calcite surfaces and endospores of *Bacillus simplex* BaSD-223 incubated for 26 weeks.

**Figure 3:**
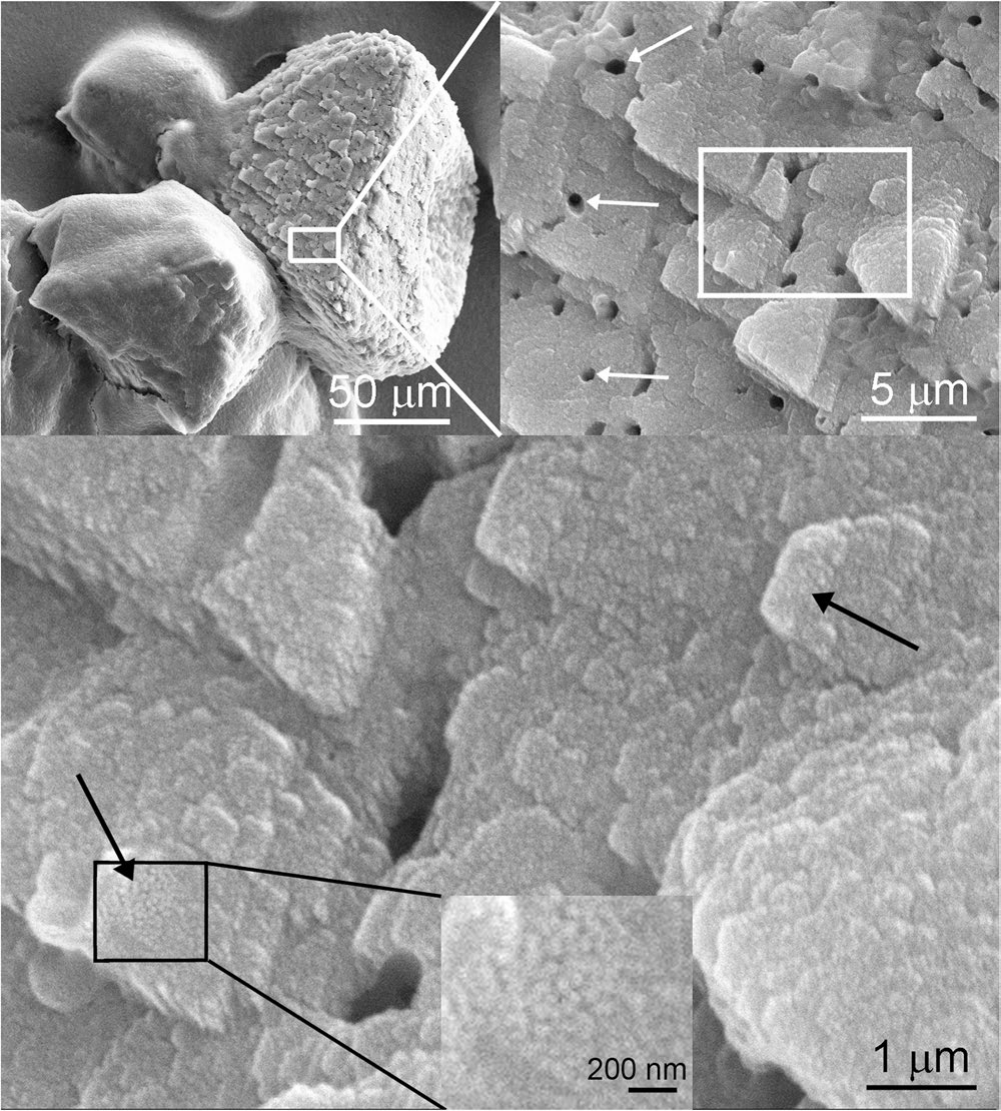
50–100 nm-sized Ca-carbonate globules (black arrow) cover calcite surface produced by *Rhodococcus degradans* BaTD–248 incubated for 26 weeks. The crystal surfaces are pitted by bacteria-shaped holes (white arrows) that presumably formed as the crystal grew around the cells, which subsequently lysed.

SEM images of *Stenotrophomonas maltophilia* BaSD-214 (Fig. 1), *Bacillus simplex* BaSD-223 (Fig. 2) and *Rhodococcus degradans* BaTD–248 (Fig. 3) colonies incubated on solid medium for 26 weeks showed rounded, triangular, square, rectangular and aggregated 5–300 micron-sized crystalline calcium carbonates. The surface of the crystals was rugged and high magnification images demonstrated they were covered by a layer that contained 1–3 micron-sized microbes and spherical 50–100 nm-sized objects (Figs. 1–3). They commonly occurred on the outer part of microbes and endospores of *Bacillus simplex* BaSD-223 (e.g., Fig. 2). In contrast, the SEM images of the negative control bacterial strains of *Staphylococcus xylosus* BaTD-289, *Herbiconiux solani* BaOD-269 and *Agromyces subbeticus* BaOD-279 did not show nano-sized globules (Supplementary Fig. S4). According to literature data, the globular and nanosize CaCO_3_ grains could correspond to ACC^2,18^. However, in our samples the 50–100 nm-size objects are presumably not individual ACC grains, but they are aggregates of EPS-covered ACC.

**Figure 4:**
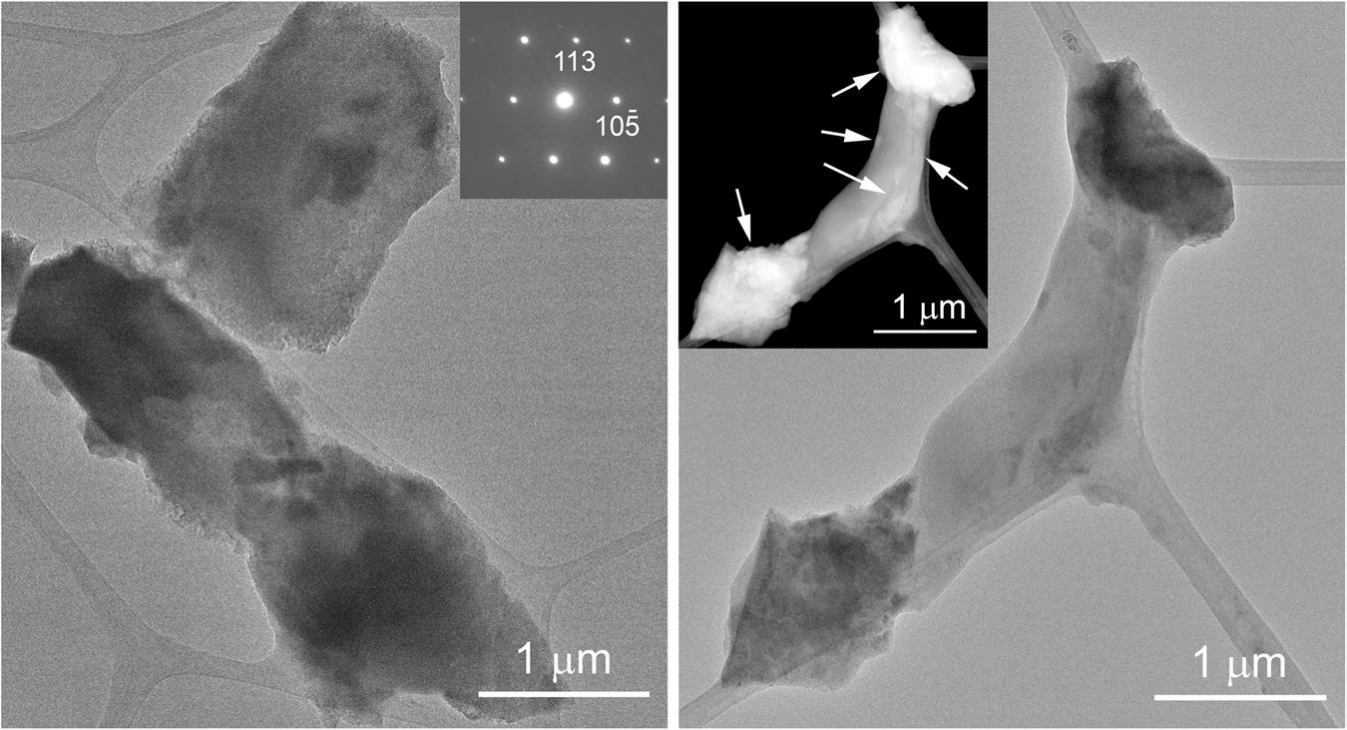
TEM images of *Rhodococcus degradans* BaTD-248 incubated for 26 weeks. The sample is dominated by well-crystalline calcite crystals, which preserve the shape of the original bacterial cells. The SAED insert shows calcite in < –58-1> projection. White arrows of the STEM image point to crystalline calcium carbonate.

In order to provide insights into the nanostructure of the precipitates, we investigated the cells of bacterial colonies incubated for 26 weeks using TEM. Since all of them developed similar precipitates according to SEM and FTIR spectroscopy (see section *Raman and FTIR investigation of bacterial carbonate precipitates*) measurements, we selected to study the nanostructure of the precipitates from the *Rhodococcus degradans* BaTD–248. Its TEM study revealed abundant micron-sized calcite grains, which preserved the morphology and size of previous bacterial cells, and some microbe remnants with crystalline CaCO_3_ coverage (Fig. 4). Globular and nanosize calcium carbonate grains were not apparent on TEM images, which observation could indicate the 26 weeks old sample batch was dominated by crystalline material and the globular grains represented only a small portion.

### Long-time stable ACC

**Figure 5:**
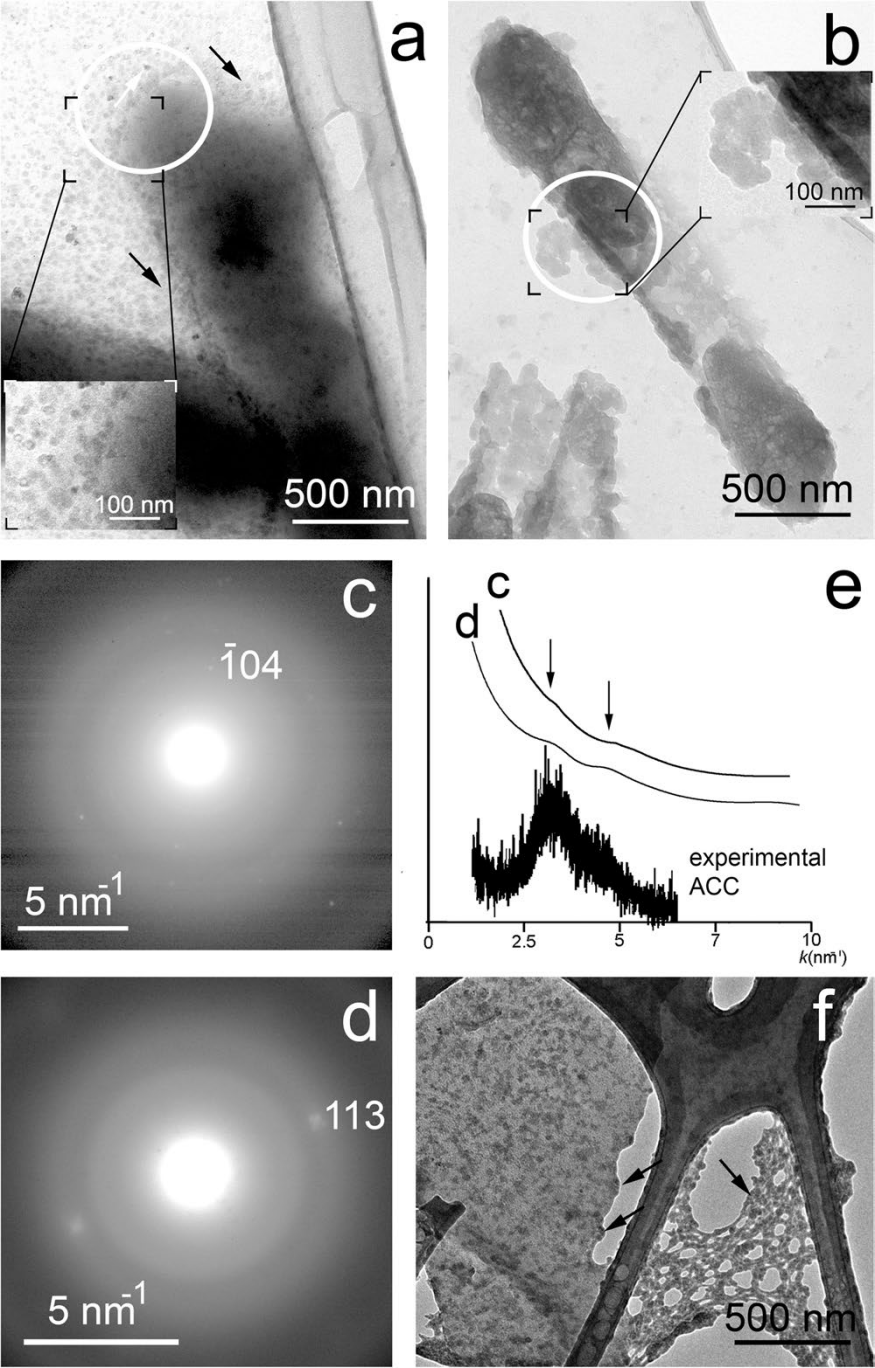
Abundant ACC forms on the surface of the bacterial cells after a few days of incubation. (**a**) TEM image of 20 nm-sized ACC (black arrows) from *Rhodococcus degradans* BaTD-248 incubated for 3 days. Although the cell surface is dominated by pale gray rounded ACC, idiomorphic dark contrast grains also occur (white arrow). (**b**) TEM image of 50 nm-sized ACC from *Rhodococcus degradans* BaTD-248 incubated for 21 days. (**c**) and (**d**) SAED patterns taken from the white circle area of (**a)**, and (**b**), respectively, show the characteristic diffuse rings of ACC and faint calcite reflection. (**e**) Distributions calculated from (**c)**, and (**d**) following the method described by Lábár^60–62^ and using the ProcessDiffraction software (version 8.7.1.; https://www.energia.mta.hu/~labar/ProcDif.htm, free program) resemble the diffraction characteristic of literature ACC. (**f**) The bacteria-produced rounded 20 nm-sized ACC is similar to the ACC (black arrows) precipitated from the drip water of Baradla Cave during 24 hours.

In order to follow the calcium carbonate precipitation process of bacterial strains, we measured colonies of *Rhodococcus degradans* BaTD-248 incubated for 3 and 21 days using TEM (Fig. 5). The investigation showed the preponderance of spherical 20 and 50 nm-sized CaCO_3_ grains in the 3 (Fig. 5a) and 21 (Fig. 5b) days samples. The grains occurred in the surroundings of the short filaments composed of rod-shaped cells. Although the 50 nm-sized grains were correlated with the colony incubated for 21 days, we presumed this colony also consisted of microbes at early stages of growth because 20 nm-sized CaCO_3_ grains occurred as well.

The SAED (selected-area electron diffraction) patterns of the spherical grains revealed the characteristic diffraction features of ACC, i.e., poorly resolved diffuse rings (Fig. 5c,d). Some faint calcite diffraction spots also occurred, which indicated a small (~5 vol%) crystalline portion besides ACC. The morphology and diffraction features (Fig. 5e) of the bacteria-produced ACC were similar to those found in the fresh precipitates of Baradla Cave (Fig. 5f), where the ACC was presumably associated with microbial activity as reported by Demény *et al*. in 2016^25^.

**Figure 6:**
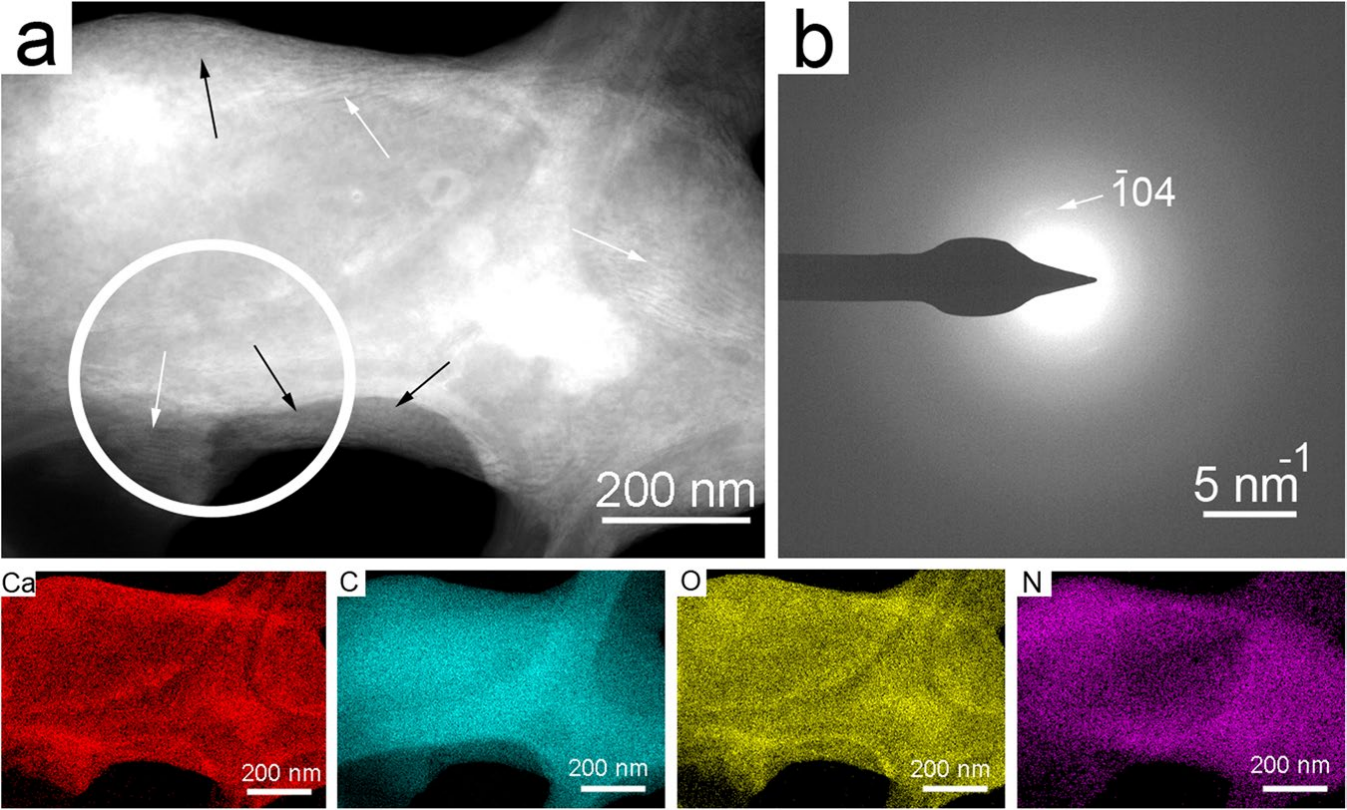
Bacterial EPS preserves ACC for at least 26 weeks. (**a**) STEM image of *Rhodococcus degradans* BaTD-248 incubated for 21 days and observed 26 weeks later. Spherical ACC grains (black arrows) and partly ordered columnar objects (white arrows) occur on the surface of the bacterial cell. (**b**) SAED pattern taken from the white circle area of (**a**) shows the characteristic diffuse rings of ACC and faint calcite reflections. The elemental maps show Ca enrichment and N decrease on the surface of the cell in consistent with ACC formation.

We studied the 21 days-old sample of *Rhodococcus degradans* BaTD-248 26 weeks later and found 20 nm-sized spherical CaCO_3_ also showing diffuse rings in consistent with ACC (Fig. 6). They dominantly occurred on the surfaces of bacterial cells. Scanning TEM images (STEM) demonstrated that a portion of the spherical grains were ordered into columnar layers (Fig. 6a) and their SAED patterns revealed faint calcite reflections (Fig. 6b). However, the major part of the grains was still untransformed ACC, indicating that the bacterial EPS could preserve this precursor form during the whole studied period.

### Raman and FTIR investigation of bacterial carbonate precipitates

To study the structure of bacteria-produced calcium carbonate crystals (Figs. 1–3), we applied Raman and FTIR spectroscopy on selected grains. We compared the data with negative control, non-carbonate precipitating, bacterial strains of *Staphylococcus xylosus* BaTD-289, *Herbiconiux solani* BaOD-269 and *Agromyces subbeticus* BaOD-279 (Supplementary Fig. S5) as well as a soda straw from Baradla Cave. Despite the grains were covered by EPS and intense fluorescence arising from this organic substance obscured the Raman signal, we could obtain appreciable data for *Stenotrophomonas maltophilia* BaSD-214.

**Figure 7:**
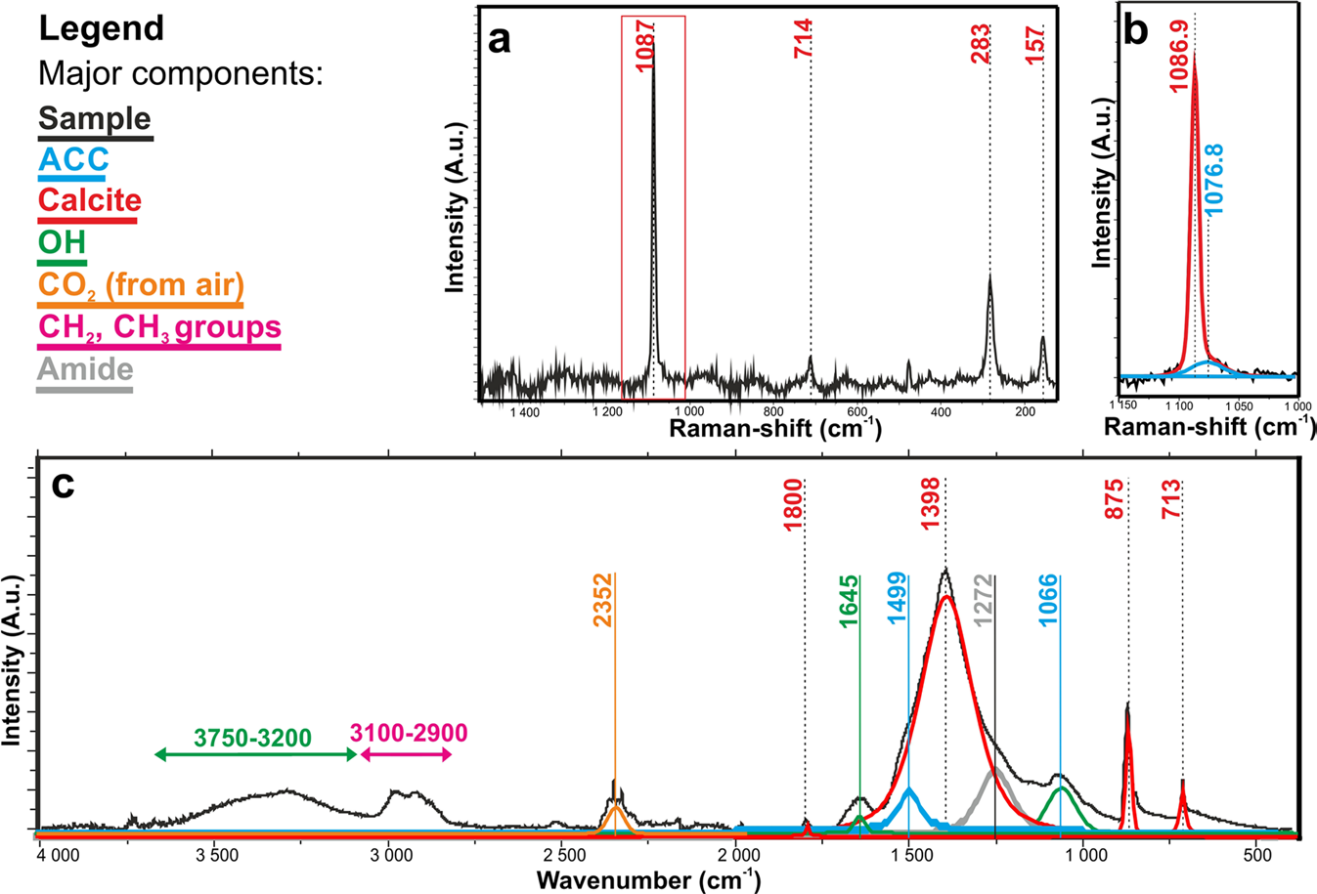
Vibrational spectroscopy indicates ACC fraction in bacterial EPS. (**a**) Baseline-corrected Raman of *Stenotrophomonas maltophilia* BaSD-214 shows calcite bands dominantly. (**b**) Deconvoluted ν_1_ band of carbonate group reveals ACC content besides calcite. (**c**) FTIR spectra of *Stenotrophomonas maltophilia* BaSD-214 EPS shows the main components (amino, methyl and hydroxyl groups), calcite and small amount of ACC.

The baseline-corrected Raman spectrum (raw data is reported in Supplementary Fig. S6) of a selected calcium carbonate grain was dominated by the Raman active bands of calcite (Fig. 7a). The most intense band, belonging to the symmetric stretching vibration (ν_1_) mode of the carbonate groups, occurred at ~1087 cm^−1^. The band had an asymmetric broadening to the lower wavenumbers, its deconvolution resulted in the appearance of a small band at ~1077 cm^−1^ (Fig. 7b), which is commonly associated with ACC^2,6,36–38^. The asymmetric stretching mode (ν_3_), the most intense band in the IR spectrum occurring at ~1400 cm^−1^, (expected to be low in intensity in the Raman spectrum) could not be detected. From the low-intensity out-of-plane and the in-plane bending modes (ν_2_ and ν_4_) of calcite, occurring at ~800 cm^−1^ and at ~712 cm^−1^, respectively, we could positively identify ν_4_ only. However, the Raman spectrum showed the calcite lattice modes in the far IR region, at 283 cm^−1^ and at 157 cm^−1^.

The calcium carbonate crystal covering EPS of *Stenotrophomonas maltophilia* BaSD-214 was evidenced by FTIR measurement. In particular, the FTIR spectrum (Fig. 7c) showed a wide band between 3700 and 3200 cm^−1^, characteristic for hydroxyl and amino groups, and they could be associated with alcohols, sugars, carboxylic, nucleic and amino acids. The characteristic amide band at 1260 cm^−1^ also occurred. The intensity ratios of CH_2_ asymmetric (2920 cm^−1^) and symmetric stretching bands (2870 cm^−1^) as well as the CH_3_ band (2956 cm^−1^) indicated the occurrence of long-chain hydrocarbon derivatives (lipids, sugar alcohol/acid esters).

The FTIR spectrum showed a wide complex band system between 1600 and 1000 cm^−1^, which we attributed to calcite and some bands related to organic material (Fig. 7c). The deconvolution of the complex band system resulted in bands at 1494 cm^−1^ and at 1066 cm^−1^, which could be associated with ACC according to literature data^6,36,37^. However, it is likely that in the mid IR region the carbonate bands of calcite and ACC coincided with numerous organic group vibrations^39^ such as CH_2_ and CH_3_ bending modes of proteins (e.g., alanine: 1465 cm^−1^; valine: 1450 cm^−1^), the C = N (imidazole ring) vibration of nucleic acids (1578 cm^−1^), ribose C-O stretchings of DNA and RNA (1100-1000 cm^−1^), stretching and bendings of uracil rings in RNA (~1000 cm^−1^) and the symmetric and asymmetric stretching mode of glutamic acid (1560 cm^−1^) and carboxylate groups (1415 cm^−1^). These organic bands are evident in the negative control samples (Supplementary Fig. S5).

The FTIR spectra of selected calcium carbonate grains from *Bacillus simplex* BaSD-223 and *Rhodococcus degradans* BaTD-248 were similar (Supplementary Fig. S5) to *Stenotrophomonas maltophilia* BaSD-214. For all three strains the carbonate bands were consistent with calcite and ACC. Since all contained the same carbonate bands and matching FTIR bands related to organic material, we propose the functional groups relevant in calcium carbonate precipitation were identical.

A question could arise about the quantity of ACC in comparison to calcite. Using the method and the calibration curves reported by Hodson *et al*.^40^, the calculated intensity ratios of ν3/ ν4 and ν2/ ν4 result in 1:4 and 1:10 ACC/calcite values for FTIR data of Fig. 7c, respectively. However, ACC quantification of vibrational spectra in bacterial samples faces several problems: (1) the wide (ν3) band for ACC strongly overlaps with the analogous band of calcite, (2) the EPS organic bands are close to those of carbonates (Fig. 7, Supplementary Fig. S5), (3) a reference sample, i.e., a bacterial strain with the same amount/ratio of organic content that precipitates calcite and no ACC, is not available, and (4) it is not possible to prepare “a calibration series”. In addition, the quantity of ACC depends on the age of a given colony. We suggest that the strains precipitate ACC first (1–2 weeks) and later calcite (4–6 weeks). Although the calcite surfaces will be recolonized again with new bacterial cells (Figs. 1–3), the carbonate grains of the old colonies (26 weeks) are definitely dominated by calcite. Thus, we can only provide a rough estimate that the ACC vs calcite content for the 26 weeks old colony (Fig. 7c) is presumably ~10% but it likely varies up to 20% depending on the age of the colony.

### Building blocks of the EPS

Our results showed that bacterial EPS played a key role during calcium carbonate precipitation and the preservation of ACC. The EPS is a multicomponent material, in order to characterize its main components, we performed pyrolysis-GC-MS measurements and GC-MS study of the products formed during acidic hydrolysis/methylation of EPS.

In general, the composition of EPS surrounding the bacterial cells strongly depends on the strain type and the media applied for cultivation^41,42^. *Bacillus simplex* belongs to the *‘Bacillus megaterium* group’ and is known to produce EPS of polysaccharide and polypeptide containing poly-γ-glutamic acid if grown for example on high glucose and protein containing media^43^. *Stenotrophomonas maltophilia* strains grown in LB medium produce EPS containing ~25% carbohydrates and ~50% proteins according to García *et al*.^44^ From the carbohydrate fraction of the EPS Abd-Alla *et al*. in 2018 reported galacturonic acid, glucuronic acid, xylose, ribose, maltose, lactose and glucose^42^. The EPS produced by *Rhodococcus* was described to consist mostly of polysaccharides. In particular, the water-soluble exopolymer of *Rhodococcus opacus* 89UMCS grown on LM medium contained 64.6% polysaccharides and 9.44% proteins^45^. *Rhodococcus* strains produced acidic polysaccharide with galactose, glucose, fucose, glucuronic acid, mannose, pyruvic acid and 5-amino-3,5-dideoxynonulosonic (rhodaminic) acid with variable molar ratio^46–48^. Moreover*, Rhodococcus rhodochrous* ATCC 53968 and *Rhodococcus erythropolis* PR4 grown on IB medium contained a fatty acid-containing extracellular polysaccharide that had 4.1–4.3 wt% palmitic acid content^47,48^. 2.9 wt% stearic acid was reported for *Rhodococcus erythropolis* PR4^48^. De Carvalho and da Fonseca in 2007^49^ demonstrated the positive effect of the amount of fatty acid content with more than 16 carbon atoms on hydrophobicity and biofilm-forming capacity of *Rhodococcus erythropolis* DCL14 cells.

We focused our investigation on *Rhodococcus degradans* BaTD-248 because this strain produced a large amount of EPS. Furthermore, its EPS composition has never been studied. Pyrolysis-GC/MS experiments have been performed in order to identify the main decomposition products of the EPS. With this information we can refer to the composition of the initial sample. The pyrolysis-GC/MS experiment of *Rhodococcus degradans* BaTD-248 associated EPS (EPS-detachment procedure described in the experimental section) was performed at 600 °C because all the typical biological building blocks: lipids, proteins, carbohydrates, and nucleic acids of the extracellular polymeric substance decompose below this temperature. Supplementary Fig. S7 shows the pyrogram of the volatile products releasing from EPS during the pyrolysis. The identification, the most abundant MS ions and the probable sources of the pyrolysis products are listed in Supplementary Table S2. The first group of unresolved peaks at low retention time (peak #1) corresponded to smaller molecular mass products consisting of mainly carbon dioxide and water formed by the fragmentation of many components and moisture evaporation. Among the pyrolysis products, we identified saturated hydrocarbons (peaks #2, #3, #5, #8, #9, #10, #13, #15, #18, #21, and #24) and chain-end unsaturated 1-alkene molecules (peaks #2, #3, #5, #8, #9, #10, #13, #16, #19, and #22). They derived from the fragmentation of the long-chain fatty acid parts of the lipids. Lauric acid (peak #27), myristic acid (peak #31) and palmitic acid (peak #36) also indicated the presence of fatty acid moieties in the EPS. Fatty acid amides (peaks #39, #42 and #43) were formed by reaction of fatty acid segments and amine groups of proteins. Fatty nitriles (peaks #29, #35, and #40) were obtained by dehydration reaction of fatty acid amides. We identified 1-hydroxy-propanone (peak #6), hydroxy-methyl-cyclopentenone (peak #12) and levoglucosan (peak #30) as the thermal decomposition products of carbohydrates. Aromatic compounds, as toluene (peak #7) and phenol (peak #14), as well as nitrogen-containing aromatic products, as benzylnitrile (peak #17), indole (peak #23), and methylindole (peak #25) were presumably the decomposition products of amino acid units of proteins especially of phenyl alanine, tyrosine and tryptophan. Peak #44 was attributed to proline-pyroglutamine, which is a representative of 2,5-diketopiperazine molecule, a cyclic dipeptide formed from the amino acid residues during the pyrolysis. In summary, the main pyrolysis products were derived from lipids, followed by amino acids, and smaller amounts of compounds are formed from carbohydrates and DNA.

In order to determine the long-chain carbon components of the studied EPS, we investigated the methylated products using GC-MS (Supplementary Figs. S8 and S9). A typical long-chain fatty acid was identified as methyl palmitate (Supplementary Fig. S9c). The MS data indicated a series of fragments with monotonically decreasing intensities with m/z = 14 mass difference correlates well with gradual CH_2_ group loss. The spectra also showed a peak at m/z = 59, which could arise from the CH_3_ group (m/z = 15) fragmented McLafferty ion (CH_3_OCH(=CH_2_)OH^+^, m/z = 74). This fragmentation product is typical for long-chain carboxylic acid methyl esters, from which we think the EPS consisted lipids that had long-chain linear, saturated and aliphatic carboxylic acid components such as palmitic acid, which is known for *Rhodococcus* spp.^49^

### Bacteria-induced ACC formation

**Figure 8:**
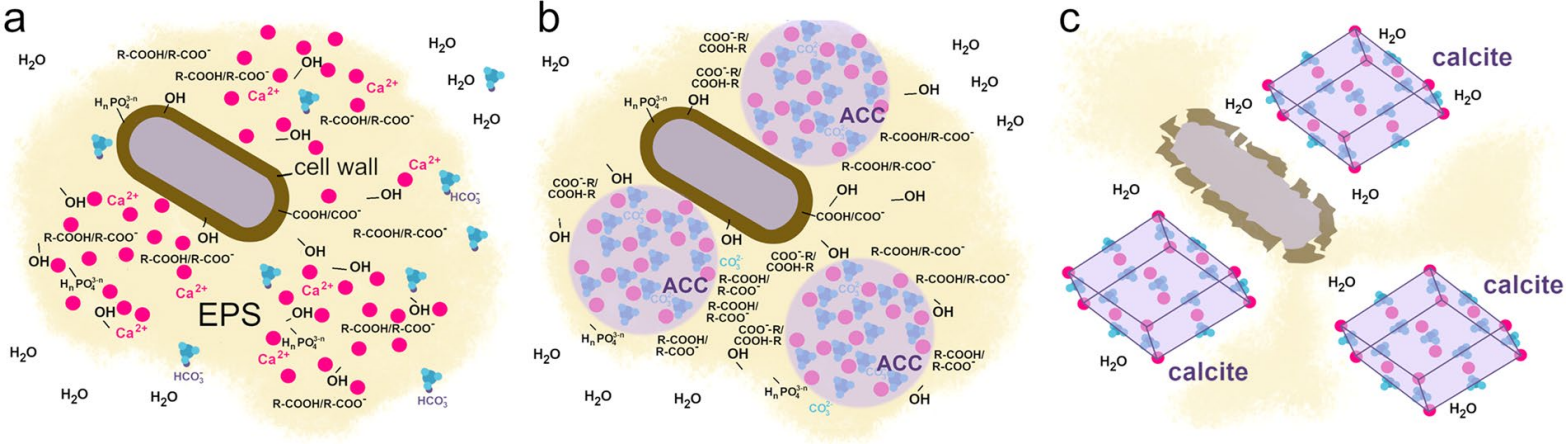
Schematic representation of the proposed model for ACC and calcite nucleation on the surface of bacterial cells. (**a**) The cell is surrounded by negatively charged ions/polarized molecules (e.g., OH, R-COOH, R-COO^-^, H_n_PO_4_^3-n^ n = 0–2) in the cell wall and EPS, which bind Ca^2+^ ions. CO_2_ – which is originated from the air or bacterial metabolic activity – dissolved in the matrix as hydrogencarbonate ion, and the bacterial metabolic processes give rise to alkaline pH and create conditions favorable for CaCO_3_ precipitation (releasing of calcium ions and formation of carbonate ions). (**b**) Firstly ACC forms, which is stabilized by the apolar hydrocarbon chain of the EPS components (R) and thus protected from water infiltration. (**c**) Gradual disruption of the EPS following cell death and partial disruption of the cell wall opens up the ACC covering hydrophobic layers and gives rise to the formation of calcite.

Our study provides new insights into the underlying process of bacteria-induced calcium carbonate crystallization (Fig. 8). After the inoculation of the B4 media, bacterial cells start to hydrolyze the yeast extract-originated amino acids, produce ammonia, increase the pH and bind the culture-medium originated Ca^2+^ to the negatively charged and polarized functional groups present on bacterial surfaces (Fig. 8a). As the pH increases (>8)^50^, the HCO_3_^-^ ions get deprotonated and the CO_3_^2-^ content increases in the EPS matrix. As a result of poor buffering capacity of the remaining yeast extract in B4 medium^50^, the pH increases gradually by bacterial activity and CaCO_3_ precipitates in the EPS (Fig. 8b). Interestingly, in these high water-containing mucoid bacterial colonies the firstly precipitating calcium carbonate is amorphous, which is in striking contrast to literature data that suggests ACC transforms within seconds in aqueous environment^1,2^. Therefore, we presume that bacterial EPS shields ACC from hydration-mediated crystallization. Within the porous three-dimensional architecture of the EPS surrounding bacterial cells distinct hydrophilic and hydrophobic microenvironments can develop. In particular, the hydrated EPS components such as polysaccharides, proteins and DNA are located along the water channels^15,44,49,51^, whereas the vicinity of lipid-like materials is hydrophobic because they have long apolar hydrocarbon chain. We presume that ACC accumulates in the water free sites, where the apolar parts of EPS components inhibit water infiltration (Fig. 8b). We identified large amount of long-chain linear fatty acid containing components in the EPS of *Rhodococcus degradans* BaTD-248. Such acid derivatives have long apolar and hydrophobic hydrocarbon chains, which might cover the ACC surface, host ACC in a “micella-like formula” and thus protect it from crystallization. In the stationary phases of the microbial growth cycle (~2 weeks after inoculation) both lysed and living bacteria occur together in the culture. As the bacterial cells die and their cell wall structure partially disintegrate, the uncontrolled enzyme release results in the local degradation of the EPS matrix and also the disintegration of the ACC covering hydrophobic layers. As the ACC grains are no longer protected, the water infiltration provides favorable conditions for the transformation of ACC to calcite (Fig. 8c).

## Conclusions and implications

Microbial activity plays an important role in calcium carbonate precipitation, on the one hand microbes change locally the physico-chemical parameters in their environment and on the other hand bacterial surfaces provide sites for nucleation and growth. In this study we isolated three cultivable heterotrophic microbial strains from the speleothems of Baradla Cave, Hungary to study CaCO_3_ precipitation, in particular ACC formation and crystallization, *in vitro*. TEM images of the colonies incubated for 3 and 21 days revealed the preponderance of spherical 20 and 50 nm-sized CaCO_3_ grains, which diffraction features were consistent with ACC, occurring in the EPS matrix. Their morphology and diffraction features were similar to those found in the fresh precipitates of Baradla Cave, which implied the ACC reported by Demény *et al*.^25^ were presumably associated with microbial activity. TEM investigation of the 21-days incubated colony measured 26 weeks later showed evidence for ACC. Its occurrence was also confirmed by Raman and FTIR data. We estimated that the ACC content compared to calcite was presumably ~10%, however it likely varies up to 20% depending on the age of the colony. The finding implied that bacterial EPS could preserve this precursor for long time even in aqueous environment and at ambient temperature, which was in strike contrast to laboratory-produced ACC. We studied the ACC-shielding EPS and confirmed that it consisted of all the typical biological building materials: lipids, proteins, carbohydrates (polymer sugar derivatives) and nucleic acids. We found large amount of long-chain fatty acid derivatives as main components. Based on the findings we proposed that (1) ACC could be enclosed in a micella-like formula of EPS components that inhibited water infiltration and (2) the crystallization of ACC could be strongly related to the lysis of bacterial cells and the disintegration of the shielding EPS.

We presume the occurrence of ACC in caves is directly linked to bacterial activity and EPS-preserved ACC is common in the carbonate precipitates of temperate zone caves. However, we expect the formation of crystalline carbonates and no ACC in caves, where bacterial activity is scarce. This expectation is supported by recent investigation from Obstanser Eishöhle. In this ice cave the low temperature (1 °C) could promote the formation and preservation of ACC, however, the TEM measurement of the fresh carbonate precipitates from Obstanser Eishöhle shows the occurrence of an aragonite crystalline precursor but no evidence for ACC^52^. The low temperature presumably makes the ice cave unfavorable for extensive microbial activity; bacterial-produced ACC could not form and the sediments are dominated by inorganic carbonates. An implication of bacterial-produced and EPS-protected ACC is that it could aid toward distinguishing abiotic and biotic carbonate precipitates in cave sediments.

The formation and month-scale preservation of ACC has further implications in speleothem research. Calcite and ACC have different stable isotope fractionations relative to water (see Demény *et al*.^25^). Fast transformation of ACC to calcite – as observed in laboratory experiments – would allow the freshly precipitated carbonate to approach the stable isotopic calcite-water equilibrium with the dripping water. On the other hand, persistence of ACC and its enclosure in the precipitating carbonate may either preserve the stable isotope compositions of ACC, or during re-crystallization the carbonate structure may become open to interactions with later infiltrating solutions that can change the original compositions of the carbonate. As a result, the ACC to calcite recrystallization can significantly affect the climatic and environmental interpretation of the chemical and isotopic signals recorded in speleothems.

## Experimental section

### Isolation of bacterial strains, phylogenetic and phenotypic analysis

Strains BaSD-214, BaSD-223, BaTD-248, BaOD-269, BaOD-279 and BaTD-289 were isolated from water samples collected in Baradla Cave, Hungary (N48°28′ E020°30′). The cave represents a UNESCO World Heritage Listed Site. Dripping water samples were collected into sterile glasses from the cave areas that were inaccessible to tourists at Nehéz-út branch. Following serial dilutions, spread plate method using calcium acetate (2.5 g/L), glucose (5 g/L), yeast extract (4 g/L) and agar-agar (20 g/L) containing B4 agar medium (modified after Boquet *et al*.^53^) was applied. After two weeks of incubation at 21 °C, the colonies were isolated and maintained as pure cultures of strains.

The strains were identified based on their partial 16S rRNA gene sequences. Following the extraction of the genomic DNA with sterile glass beads in a mixer mill [Retsch Oscillating Mill MM400 (Retsch, Haan, Germany)], 16S rRNA genes were amplified by polymerase chain reaction using 27 forward (5′-AGA GTT TGA TCM TGG CTC AG -3′)^54^ and 1401 reverse (5′-CGG TGT GTA CAA GAC CC - 3′)^55^ bacterial primers following the protocol described by Krett *et al*. in 2017^56^. The 16S rRNA genes were sequenced with automated Sanger-method using 27 forward primer at the LGC Genomics (Berlin, Germany). Manual correction of the chromatograms was carried out with Chromas software (Technelysium Pty Ltd., Australia; version 2.6.6.; http://technelysium.com.au/wp/chromas/) and partial 16S rRNA gene sequences were compared with EzBioCloud database entries^57^.

Metabolic activity tests of strains BaSD-214, BaSD-223 and BaTD-248 were applied according to the procedures described by Barrow and Feltham in 2003^58^. We performed hydrolysis of casein, starch, gelatin, DNA, Tween 80, Barritt’s Voges-Proskauer tests, production of hydrogen sulphide from cysteine, NH_3_ production from peptone, Baird-Parker’s phosphatase activity, urease activity, nitrate reduction to nitrite and ammonia or nitrogen as well as we performed the Hugh–Leifson’s oxidation-fermentation test of D-glucose^59^. API 50CH (bioMérieux) test was used to determine carbon sources utilization according to the manufacturer’s instructions. The API test results were recorded after 3 days incubation at 28 °C.

### Calcium carbonate precipitation experiments and characterization methods

Calcium carbonate precipitation capability of bacterial strains was investigated on B4 solid and B4 liquid medium (without agar-agar). The pH of the medium was adjusted to 7.5. The B4 agar plates were inoculated in 3 parallel cross shapes and sealed with Parafilm to prevent rapid dehydration. 5 ml liquid media were inoculated with one loopful of bacteria in triplicates. They were incubated for weeks at 21 °C and periodically examined by light microscopy to reveal the presence of the precipitates.

For the scanning electron microscopy (SEM) analysis, desiccated cultures on agar blocks were mounted on sample holders and coated with palladium. These samples were measured using a Zeiss EVO 40 scanning electron microscope operated at 20 kV. A few bacterial cells were mixed with bidistilled water and three droplets were dried on lacey-C–coated Cu (Agar Scientific) transmission electron microscopy (TEM) grids. For TEM investigation we used a 100 keV Morgagni268D, a 200 KeV Philips CM20 and a 200 keV Thalos Thermo Scientific transmission electron microscope. Radial distributions of intensities were generated from SAED patterns following the method described by Lábár^60–62^ and using the ProcessDiffraction software (version 8.7.1.; https://www.energia.mta.hu/~labar/ProcDif.htm, free program).

Fourier transform attenuated total reflectance infrared spectroscopy (FTIR-ATR) measurements were performed on bacterial samples (4000 and 400 cm^−1^, 32 scans) using a Tensor 27 Bruker IR spectrometer with an A225 Platinum ATR unit consisting of a diamond crystal (refractive index: 2.4). The background signal was measured in air. We performed Raman scattering measurements at room temperature (1800-100 cm^-1^, Renishaw 1000 micro-Raman spectrometer, 2 cm^−1^ spectral resolution). The excitation wavelength was 532 nm (laser diode) and the spectra evaluations were performed by using the Labspec5 program. For the deconvolution of the overlapping bands the line shape of pseudo-Voigt functions was fit after the polynomial baseline correction. The fitting method was optimized automatically by the least-square algorithm.

### EPS extraction and characterization methods

All the used chemicals (solvents, reagents) were supplied by Deuton-X Ltd. To detach bacterial-associated EPS, the strains were grown on B4 agar medium for 5 days at 21 °C. Approximately 1.5 g bacterial biomass was collected using a metal spatula and suspended in distilled water and vortexed rigorously. EPS was removed from the cells by double centrifugation at 10000 g for 15 minutes. The resulting liquid phase was treated with twice the volume of cold absolute ethanol. The ethanol solution was added drop by drop under constant stirring and the sample was stored at 4 °C overnight in order to enhance the EPS precipitation. The precipitated EPS was collected by centrifugation at 12000 g for 10 minutes at 4 °C and desiccated at 48 °C in a thermostat incubator.

We pyrolyzed ~1 mg extracellular polymeric substrate at 600 °C for 20 s in inert atmosphere (helium) using a Pyroprobe 2000 pyrolyzer coupled to an Agilent 6890 A/5973 gas chromatograph (GC)/mass selective detector. The pyrolyzer chamber and the injector of the GC were heated to 280 °C. The injector operated with split ratio of 1:20. The formed compounds were separated on a capillary column (type: DB-1701; 30 m × 0.25 mm, 0.25 μm film thickness; 40 °C (4 min)–6 °C min^-1^ - 280 °C (7 min)). The mass range of *m/z* 14–550 was scanned by the mass spectrometer (MS) which was operated in electron impact mode (70 eV). For identification we used the NIST11 mass spectral library.

In order to determine the long-chain carbon components of the studied EPS, we performed acidic hydrolysis, followed by ethereal extraction of organic acids and methylation with diazomethane at room temperature. We investigated the methyl-ester products using a Shimadzu QP2010 GC-MS instrument. Accurately 5 μl solution was injected to the 300 °C heated injector, which was operated in split mode with the split ratio of 1:300. Solvent delay was applied for 2.3 min. The products were separated on a RxiR-5SIL MS capillary column (30 m × 0.25 mm, 0.25 μm film thickness). The oven of the GC was programmed to hold at 70 °C for 1 min then increase the temperature at a rate of 20 °C min^-1^ to 340 °C (hold for 1 min). The mass range of *m/z* 10 − 800 was scanned by the MS in electron impact mode at 70 eV electron energy. The identification of the compounds was based on different mass spectral libraries.

## Supplementary information

Supplementary information.

## Data Availability

The 16S rRNA gene sequences of the strains BaSD-214, BaSD-223, BaTD-248, and BaOD-269, BaOD-279 and BaTD-289 were deposited in the GenBank/ENA/DDBJ database under the following accession numbers: MK881132-MK881134 and MT373569-MT373571, respectively. All data generated or analysed during this study are included in this published article (and its Supplementary Information file).

## References

[1] Weiner S, Sagi I, Addadi L (2005). Choosing the crystallization path less traveled. Science.

[2] Konrad F, Gallien F, Gerard DE, Dietzel M (2016). Transformation of amorphous calcium carbonate in air. Cryst. Growth Des..

[3] Aizenberg J, Lambert G, Weiner S, Addadi L (2002). Factors involved in the formation of amorphous and crystalline calcium carbonate: a study of an ascidian skeleton. J. Am. Chem. Soc..

[4] Raz S (2003). The transient phase of amorphous calcium carbonate in sea urchin larval spicules: the involvement of proteins and magnesium ions in its formation and stabilization. Adv. Funct. Mater..

[5] Loste E, Wilson RM, Seshadri R, Meldrum FC (2003). The role of magnesium in stabilising amorphous calcium carbonate and controlling calcite morphologies. J. Cryst. Growth..

[6] Purgstaller B, Mavromatis V, Immenhauser A, Dietzel M (2016). Transformation of Mg-bearing amorphous calcium carbonate to Mg-calcite – *In situ* monitoring. Geochim. Cosmochim. Ac..

[7] Aizenberg J, Addadi L, Weiner S, Lambert G (1996). Stabilization of amorphous calcium carbonate by specialized macromolecules in biological and synthetic precipitates. Adv. Mater..

[8] Reggi M (2014). Biomineralization in mediterranean corals: the role of the intraskeletal organic matrix. Cryst. Growth Des..

[9] Gong YU (2012). Phase transitions in biogenic amorphous calcium carbonate. Proc. Natl. Acad. Sci. USA..

[10] Marxen JC, Becker W, Finke D, Hasse B, Epple M (2003). Early mineralization in Biomphalaria glabrata: microscopic and structural results. J. Mollus. Stud..

[11] Gago-Duport L, Briones MJ, Rodríguez JB, Covelo B (2008). Amorphous calcium carbonate biomineralization in the earthworm’s calciferous gland: pathways to the formation of crystalline phases. J. Struct. Biol..

[12] Shechter A (2008). Reciprocal changes in calcification of the gastrolith and cuticle during the molt cycle of the red claw crayfish Cherax quadricarinatus. Biol. Bull..

[13] Beveridge TJ, Murray RG (1976). Uptake and retention of metals by cell walls of Bacillus subtilis. J. Bacteriol.

[14] Braissant O (2007). Exopolymeric substances of sulfate-reducing bacteria: Interactions with calcium at alkaline pH and implication for formation of carbonate minerals. Geobiology.

[15] Flemming HC, Wingender J (2010). The biofilm matrix. Nat. Rev. Microbiol..

[16] Dittrich M, Sibler S (2010). Calcium carbonate precipitation by cyanobacterial polysaccharides. Geol. Soc. Spec. Publ..

[17] Cacchio P (2004). Involvement of microorganisms in the formation of carbonate speleothems in the Cervo Cave (L’Aquila-Italy). Geomicrobiol. J..

[18] Obst M (2009). Precipitation of amorphous CaCO3 (aragonite-like) by cyanobacteria: A STXM study of the influence of EPS on the nucleation process. Geochim. Cosmochim. Ac.

[19] Rusznyák A (2012). Calcite biomineralization by bacterial isolates from the recently discovered pristine karstic Herrenberg Cave. Appl. Environ. Microbiol..

[20] Lv JJ, Ma F, Li FC, Zhang CH, Chen JN (2017). Vaterite induced by Lysinibacillus sp. GW-2 strain and its stability. J. Struct. Biol..

[21] Zhang C, Lv J, Li F, Li X (2017). Nucleation and growth of Mg-calcite spherulites induced by the bacterium Curvibacter lanceolatus strain HJ-1. Microsc. Microanal..

[22] Jones B, Peng X (2012). Amorphous calcium carbonate associated with biofilms in hot spring deposits. Sediment. Geol..

[23] Diaz MR, Eberli GP, Blackwelder P, Phillips B, Swart PK (2017). Microbially mediated organomineralization in the formation of ooids. Geology.

[24] Banks ED (2010). Bacterial calcium carbonate precipitation in cave environments: a function of calcium homeostasis. Geomicrobiol. J..

[25] Demény A (2016). Formation of amorphous calcium carbonate in caves and its implications for speleothem research. Sci. Rep..

[26] Berg G, Marten P, Ballin G (1996). Stenotrophomonas maltophilia in the rhizosphere of oilseed rape - occurrence, characterization and interaction with phytopathogenic fungi. Microbiol. Res..

[27] Logan, N. A. & de Vos, P. *Bacillus* in Bergey's Manual of Systematics of Archaea and Bacteria (ed. Whitman, W. B., Rainey, F., Kämpfer, P., Trujillo, M., Chun, J., de Vos, P., Hedlund, B., Dedysh, S.) 1-164 (John Wiley and Sons, 2015).

[28] Švec P (2015). Classification of strain CCM 4446^T^ as Rhodococcus degradans sp. nov.. Int. J. Syst. Evol. Microbiol..

[29] Fang BZ (2017). Insights on the effects of heat pretreatment, pH, and calcium salts on isolation of rare Actinobacteria from karstic caves. Front. Microbiol..

[30] Neal DJ, Wilkinson SG (1982). Lipopolysaccharides from Pseudomonas maltophilia. Structural studies of the side-chain, core, and lipid-A regions of the lipopolysaccharide from strain NCTC 10257. Eur. J. Biochem.

[31] Stewart GC (2015). The exosporium layer of bacterial spores: a connection to the environment and the infected host. Microbiol. Mol. Biol. Rev..

[32] Jones, A. L. & Goodfellow, M. *Rhodococcus* in Bergey's Manual of Systematics of Archaea and Bacteria (ed. Whitman, W. B., Rainey, F., Kämpfer, P., Trujillo, M., Chun, J., de Vos, P., Hedlund, B., Dedysh, S.) 1-15 (John Wiley and Sons, 2015).

[33] Palleroni, N. J. *Stenotrophomonas* in Bergey’s Manual of Systematics of Archaea and Bacteria (ed. et al) 1–20 (John Wiley and Sons, 2015).

[34] Sanchez-Moral S (2004). Bioinduced barium precipitation in St. Callixtus and Domitilla catacombs. Ann. Microbiol..

[35] Achal V, Pan X (2011). Characterization of urease and carbonic anhydrase producing bacteria and their role in calcite precipitation. Curr. Microbiol..

[36] Andersen FA, Brecevic L (1991). Infrared spectra of amorphous and crystalline calcium carbonate. Acta Chem. Scand..

[37] Addadi L, Raz S, Weiner S (2003). Taking advantage of disorder: amorphous calcium carbonate and its roles in biomineralization. Adv. Mater..

[38] Wang D, Hamm LM, Bodnar RJ, Dove PMJ (2012). Transformation of Mg-bearing amorphous calcium carbonate to Mg-calcite – *In situ* monitoring. Raman Spectrosc.

[39] Socrates, G. Infrared and Raman Characteristic Group Frequencies (John Wiley and Sons, 2001).

[40] Hodson ME (2015). Biomineralisation by earthworms – an investigation into the stability and distribution of amorphous calcium carbonate. Geochem. Trans..

[41] Cox JS, Smith DS, Warren LA, Ferris FG (1999). Characterizing heterogeneous bacterial surface functional groups using discrete affinity spectra for proton binding. Environ. Sci. Technol..

[42] Abd-Alla MH, Bashandy SR, Nafady NA, Hassan AA (2018). Enhancement of exopolysaccharide production by Stenotrophomonas maltophilia and Brevibacillus parabrevis isolated from root nodules of Cicer arietinum L. and Vigna unguiculata L. (Walp.) plants. Rend. Lincei-Sci. Fis.

[43] Guex-Holzer S, Tomcsik J (1956). The isolation and chemical nature of capsular and cell-wall haptens in a Bacillus species. J. Gen. Microbiol..

[44] García CA, Alcaraz ES, Franco MA, Passerini de Rossi BN (2015). Iron is a signal for Stenotrophomonas maltophilia biofilm formation, oxidative stress response, OMPs expression, and virulence. Front. Microbiol..

[45] Czemierska M, Szcześ A, Pawlik A, Wiater A, Jarosz-Wilkołazka A (2016). Production and characterisation of exopolymer from Rhodococcus opacus. Biochem. Eng. J..

[46] Severn WB, Richards JC (1999). The structure of the specific capsular polysaccharide of Rhodococcus equi serotype 4. Carbohydr. Res..

[47] Urai M, Anzai H, Iwabuchi N, Sunairi M, Nakajima M (2004). A novel viscous extracellular polysaccharide containing fatty acids from Rhodococcus rhodochrous ATCC 53968. Actinomycetologica.

[48] Urai M (2007). Structural analysis of an acidic, fatty acid ester-bonded extracellular polysaccharide produced by a pristane-assimilating marine bacterium, Rhodococcus erythropolis PR4. Carbohydr. Res..

[49] de Carvalho CC, da Fonseca MM (2007). Preventing biofilm formation: promoting cell separation with terpenes. FEMS Microbiol. Ecol.

[50] Marvasi M (2012). Importance of B4 medium in determining organomineralization potential of bacterial environmental isolates. Geomicrobiol. J..

[51] Wilking JN (2013). Liquid transport facilitated by channels in Bacillus subtilis biofilms. Proc. Natl. Acad. Sci. USA.

[52] Németh P (2018). A nanocrystalline monoclinic CaCO3 precursor of metastable aragonite. Sci. Adv..

[53] Boquet E, Boronat A, Ramos-Cormenzana A (1973). Production of calcite (calcium carbonate) crystals by soil bacteria is a general phenomenon. Nature.

[54] Lane, D. J. 16S/23S rRNA sequencing in Nucleic Acid Techniques In Bacterial Systematics (ed. Stackebrandt, E., Goodfellow M.) 115-149 (Wiley, 1991).

[55] Nübel U (1996). Sequence heterogeneities of genes encoding 16S rRNAs in Paenibacillus polymyxa detected by temperature gradient gel electrophoresis. J. Bacteriol.

[56] Krett G, Szabó A, Felföldi T, Márialigeti K, Borsodi KA (2017). The effect of reconstruction works on planktonic bacterial diversity of a unique thermal lake revealed by cultivation, molecular cloning and next generation sequencing. Arch. Microbiol..

[57] Yoon SH (2017). Introducing EzBioCloud: A taxonomically united database of 16S rRNA and whole genome assemblies. Int. J. Syst. Evol. Microbiol.

[58] Barrow, G. I. & Feltham, R. K. A. Cowan And Steel’s Manual For The Identification Of Medical Bacteria (Cambridge University Press, 2003).

[59] Hugh R, Leifson E (1953). The taxonomic significance of fermentative versus oxidative metabolism of carbohydrates by Gram negative bacteria. J. Bacteriol.

[60] Lábár JL (2008). Electron diffraction based analysis of phase fractions and texture in nanocrystalline thin film, Part I: Principles. Microsc. Microanal..

[61] Lábár JL (2009). Electron diffraction based analysis of phase fractions and texture in nanocrystalline thin film, Part II: Implementation. Microsc. Microanal..

[62] Lábár JL (2012). Electron diffraction based analysis of phase fractions and texture in nanocrystalline thin film, Part III: Application Examples. Microsc. Microanal..

