# Supplementary material for: Cave bacteria-induced amorphous calcium carbonate formation
Source: Sci Rep. 2020 May 26;10:8696. doi: 10.1038/s41598-020-65667-w (PMC7251137; doi:10.1038/s41598-020-65667-w)
Supplement: Supplementary file 1 — Supplementary information. [file 41598_2020_65667_MOESM1_ESM.pdf]

Supplementary Information

Cave bacteria-induced amorphous calcium carbonate formation

Nóra Tünde Enyedi[1], Judit Makk[1], László Kótai[2,3], Bernadett Berényi[2], Szilvia Klébert[2], Zoltán Sebestyén[2], Zsombor Molnár[4], Andrea K. Borsodi[1], Szabolcs Leél-Őssy[5], Attila Demény[6] & Péter Németh[2,4]*

[1]Department of Microbiology, Faculty of Science, Eötvös Loránd University, Pázmány P. sétány 1/C, H-1117 Budapest, Hungary.

[2]Institute of Materials and Environmental Chemistry, Research Centre for Natural Sciences, Hungarian Academy of Sciences, Magyar tudósok körútja 2, H-1117 Budapest, Hungary

[3]Deuton-X Ltd., Selmeci u. 89, H-2030, Érd, Hungary.

[4]Department of Earth and Environmental Sciences, University of Pannonia, Egyetem út 10, H-8200, Veszprém, Hungary.

[5]Department of Physical and Applied Geology, Faculty of Science, Eötvös Loránd University, Pázmány P. sétány 1/C, H-1117 Budapest, Hungary.

[6]Institute for Geological and Geochemical Research, Research Centre for Astronomy and Earth Sciences, Hungarian Academy of Sciences, Budaörsi út 45, H-1112 Budapest, Hungary.

*Correspondence to: nemeth.peter@ttk.mta.hu

Contents

Supplementary Figure S1: TEM images of nanocrystalline vaterite in the control liquid sample.

Supplementary Figure S2: Light microscope images show calcium carbonate precipitates on the surface of the colonies and no precipitations in desiccated agar and the colonies of negative controls after incubation for 26 weeks.

Supplementary Figure S3: Light microscope images show the amount of calcium carbonate precipitates increases with time within the colonies of *Rhodococcus degradans* BaTD-248.

Supplementary Figure S4: SEM images of the control, non-carbonate precipitating bacterial strains.

Supplementary Figure S5: FTIR spectra of precipitated grains collected from bacterial cultures in comparison with negative controls.

Supplementary Figure S6: Raw Raman data of *Stenotrophomonas maltophilia* BaSD-214.

Supplementary Figure S7: Pyrolysis-GC-MS chromatogram of *Rhodococcus degradans* BaTD-248 associated EPS.

Supplementary Figure S8: GC-MS chromatogram of acid hydrolized/methylated products from *Rhodococcus degradans* BaTD-248 associated EPS.

Supplementary Figure S9: Representative MS spectra of long-chain carbon components.

Supplementary Table S1: Phenotypic characteristics of the bacterial isolates.

Supplementary Table S2: The main decomposition products released in the Py-GC/MS experiments from *Rhodococcus degradans* BaTD-248 associated EPS.

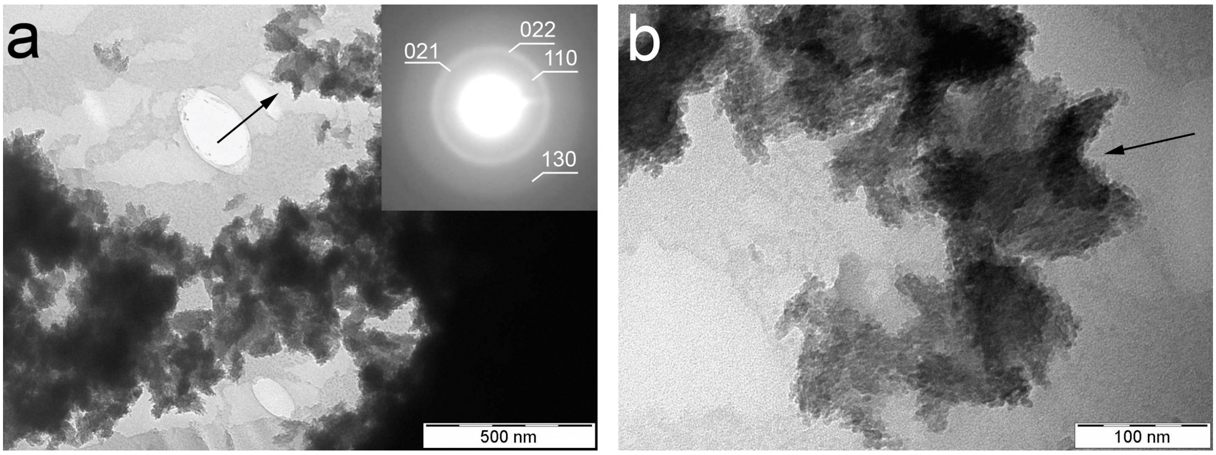

Supplementary Figure S1. TEM images show abundant nanocrystalline vaterite in the control (non-inoculated) liquid sample. a) TEM image and its corresponding ED pattern with the most intense vaterite reflections. b) Magnified area from a). Black arrows point to the same areas.

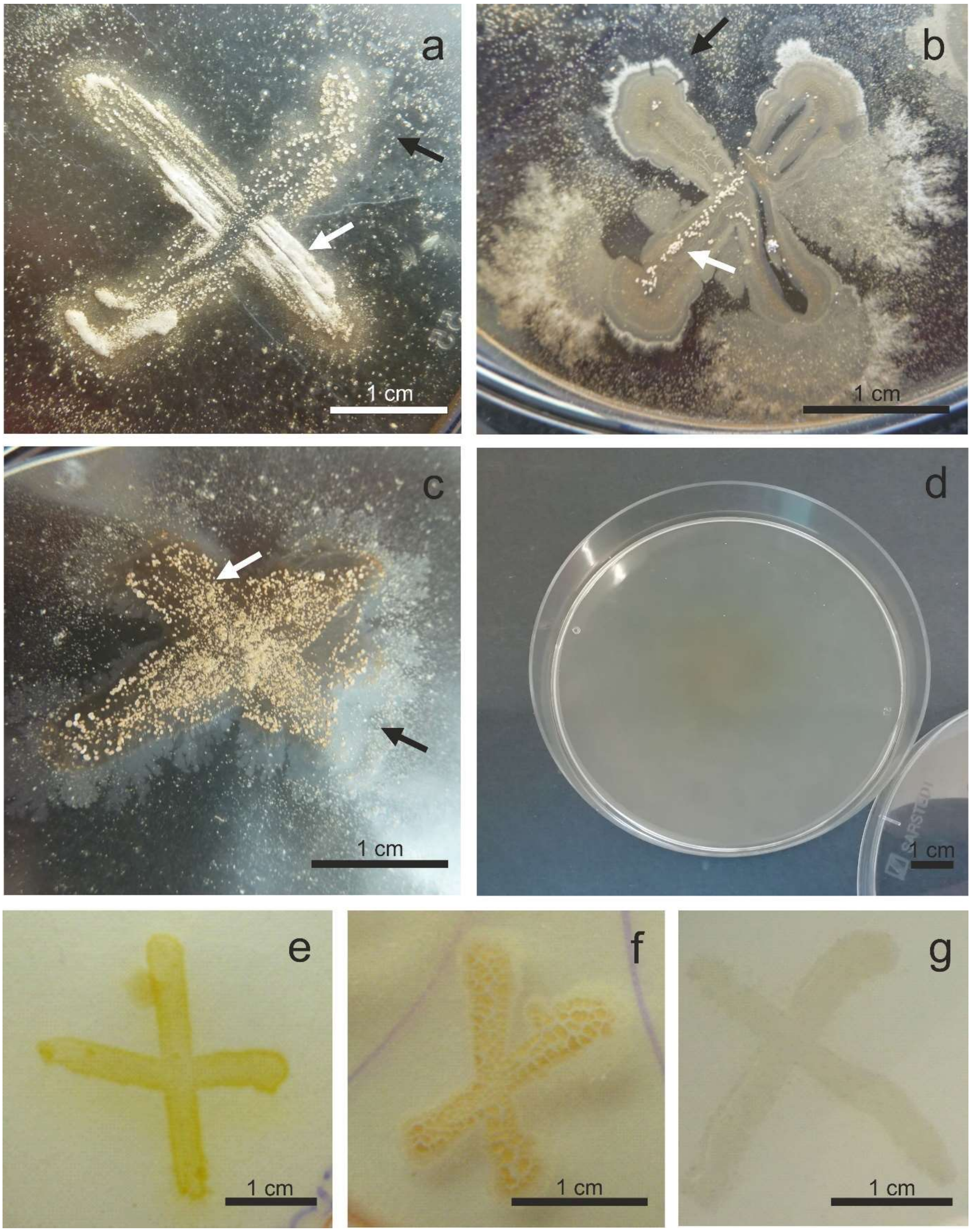

Supplementary Figure S2. Light microscope images show calcium carbonate precipitates on the surface of the colonies after incubation for 26 weeks: (**a**) *Stenotrophomonas maltophilia* BaSD-214, (**b**) *Bacillus simplex* BaSD-223 and (**c**) *Rhodococcus degradans* BaTD-248 produce visible amount of calcium carbonate in the cross shaped colonies. No precipitates are observed on the non-inoculated B4 agar plate desiccated after 26 weeks (**d**) and the colonies of the negative controls: (**e**) *Agromyces subbeticus* BaOD-279, (**f**) *Staphylococcus xylosus* BaTD-289 and (**g**) *Herbiconiux solani* BaOD-269. Black arrows point to the edges of the colonies.

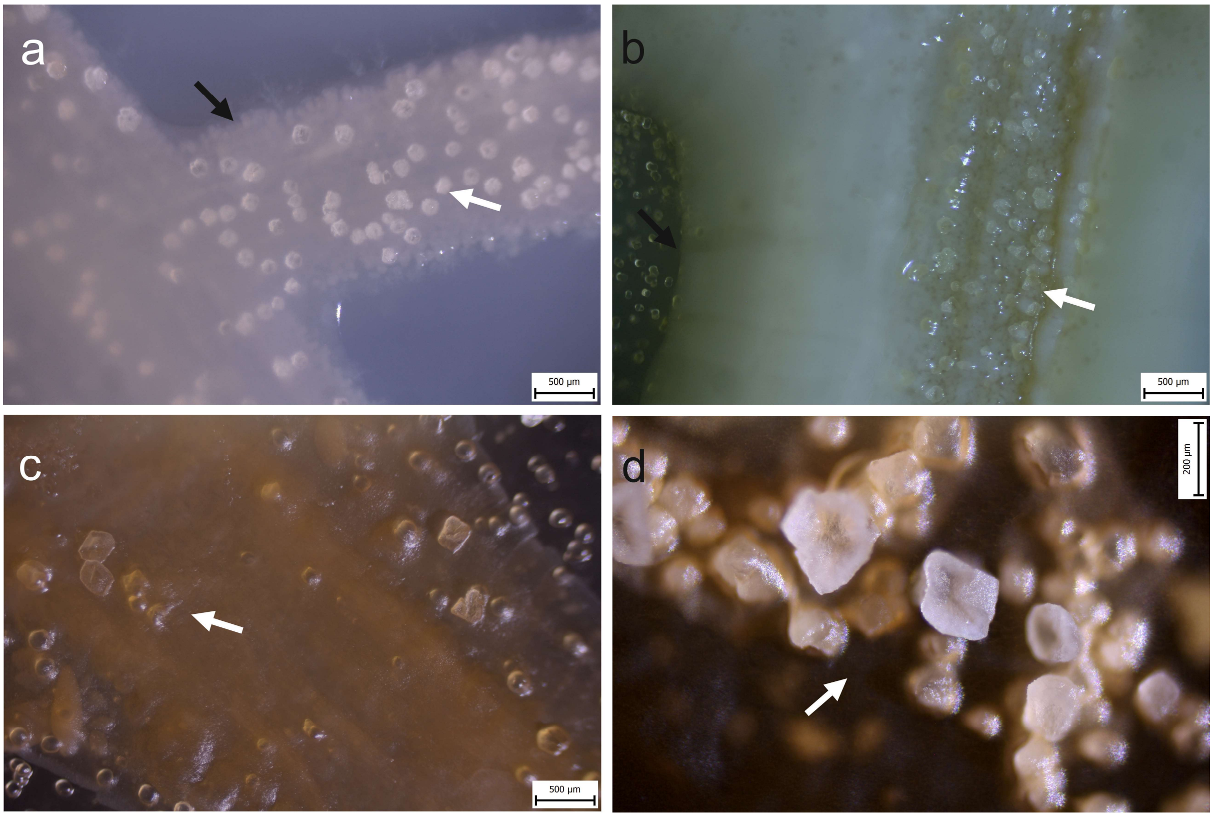

Supplementary Figure S3. Light microscope images show the amount of calcium carbonate precipitates (white arrows) increases with time within the colonies of *Rhodococcus degradans* BaTD-248. Colonies were incubated for (**a**) 2 weeks, (**b**) 4 weeks, (**c**) 8 weeks and (**d**) 22 weeks. Black arrows point to the edges of the colonies.

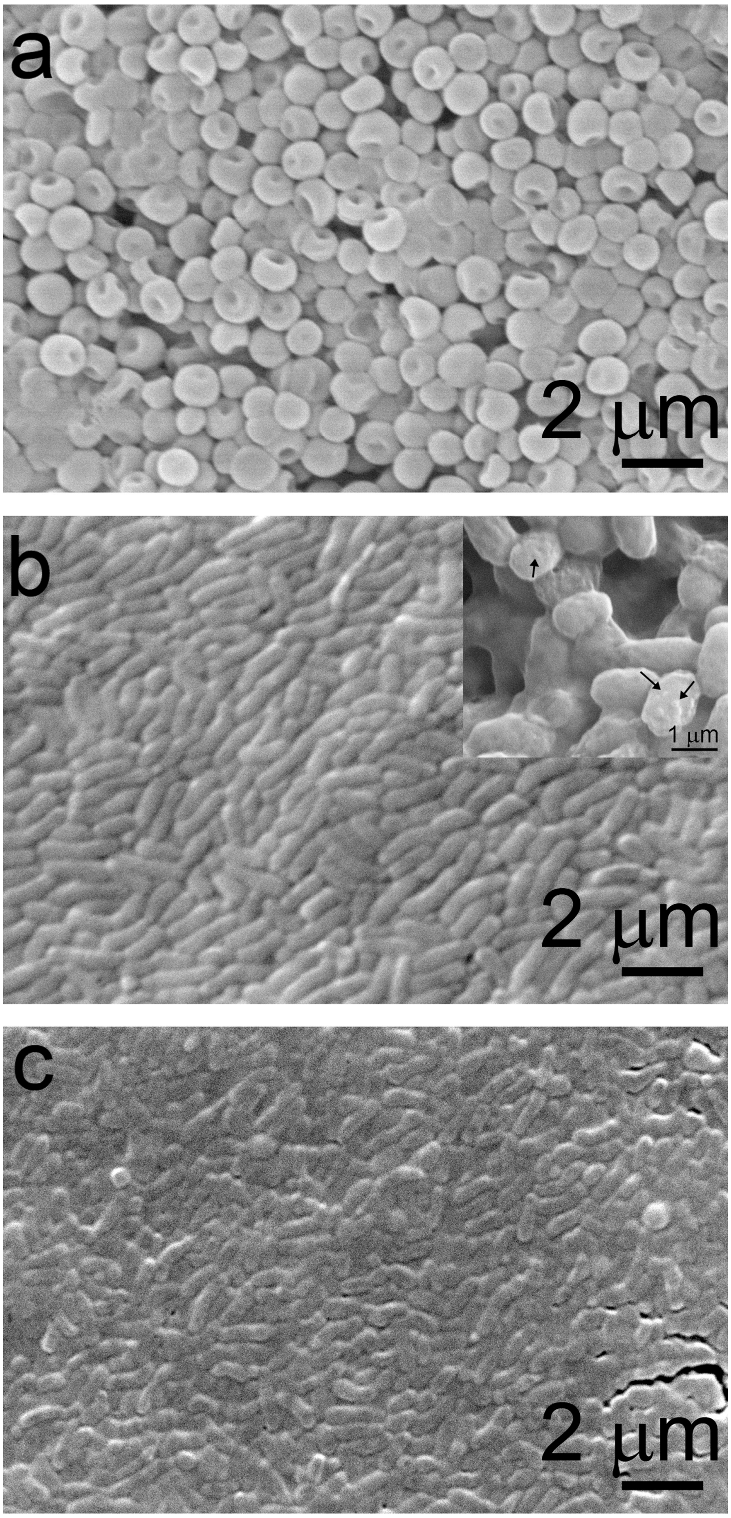

Supplementary Figure S4. SEM images of the control, non-carbonate precipitating bacterial strains of *Staphylococcus xylosus* BaTD-289 (**a**), *Herbiconiux solani* BaOD-269 (**b**), and *Agromyces subbeticus* BaOD-279 (**c**). Colonies were incubated for 8 weeks under the same conditions as the carbonate precipitating bacterial strains. Nano-sized globules are not visible on the surface of the cells of these bacterial strains. For comparison the nano-sized globules (black arrows) of *Bacillus simplex* BaSD-223 are also shown on the right upper corner of panel b.

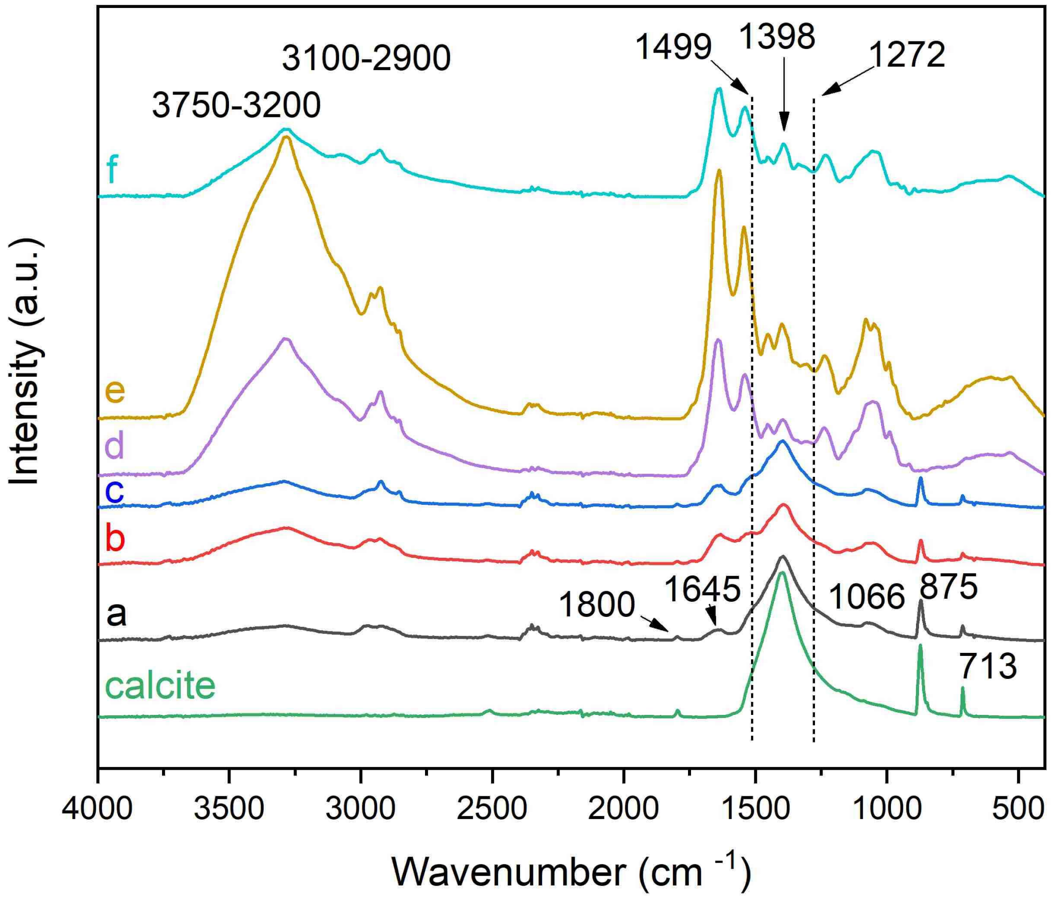

Supplementary Figure S5. FTIR spectra of precipitated grains collected from **a**) *Stenotrophomonas maltophilia* BaSD-214, **b**) *Bacillus simplex* BaSD-223, and **c**) *Rhodococcus degradans* BaSD-248 cultures incubated for 26 weeks in comparison with the EPS of negative controls **d**) *Agromyces subbeticus* BaOD-279, **e**) *Staphylococcus xylosus* BaTD-289 and **f**) *Herbiconiux solani* BaOD-269 incubated for 8 weeks. The bottom spectrum shows the characteristic calcite bands of a soda straw from Baradla Cave.

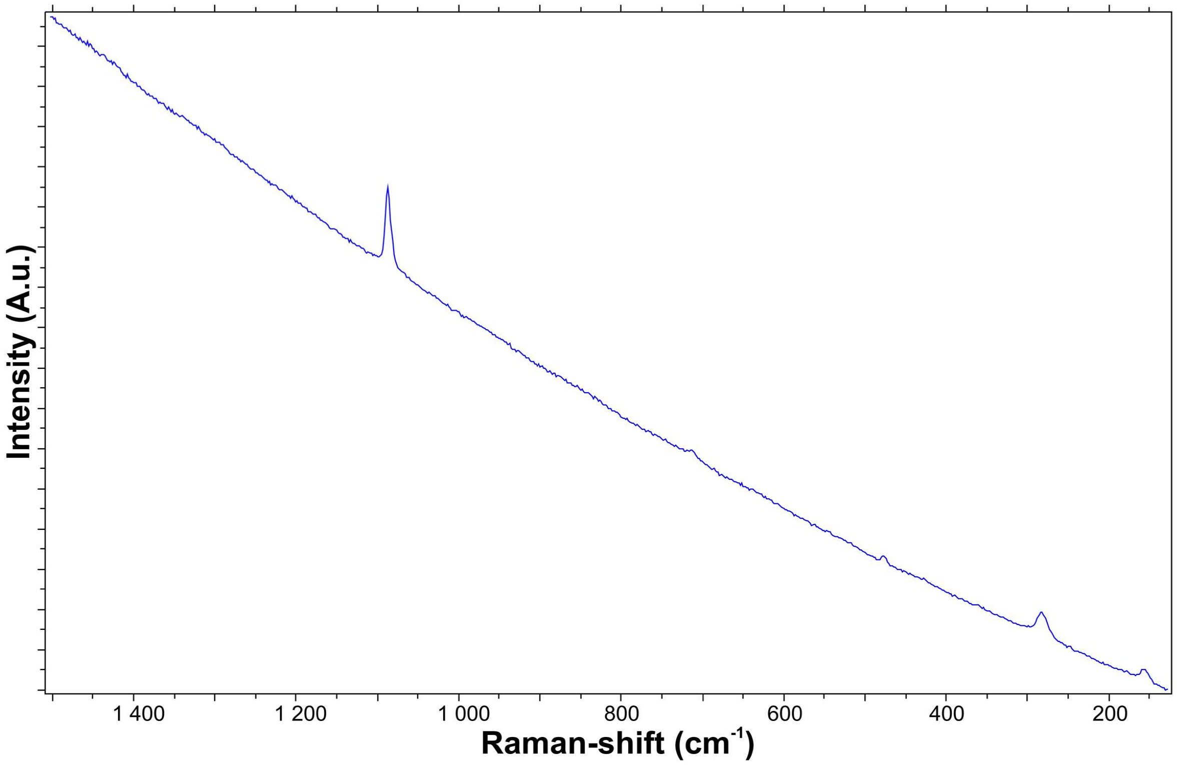

Supplementary Figure S6. Raw Raman data of *Stenotrophomonas maltophilia* BaSD-214.

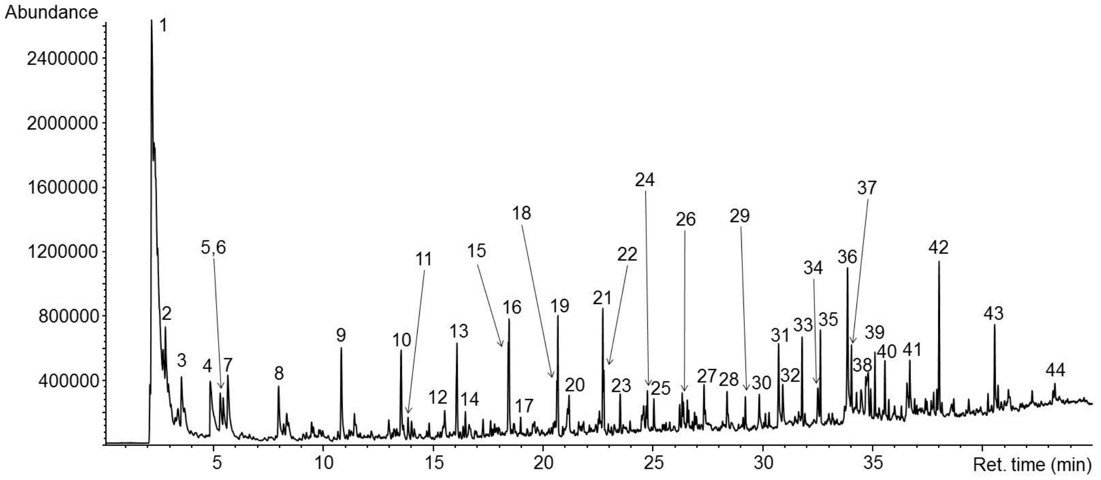

Supplementary Figure S7. Pyrolysis-GC-MS chromatogram of *Rhodococcus degradans* BaTD-248 associated EPS measured at 600 °C. Numbered peak identities are given in Table S2.

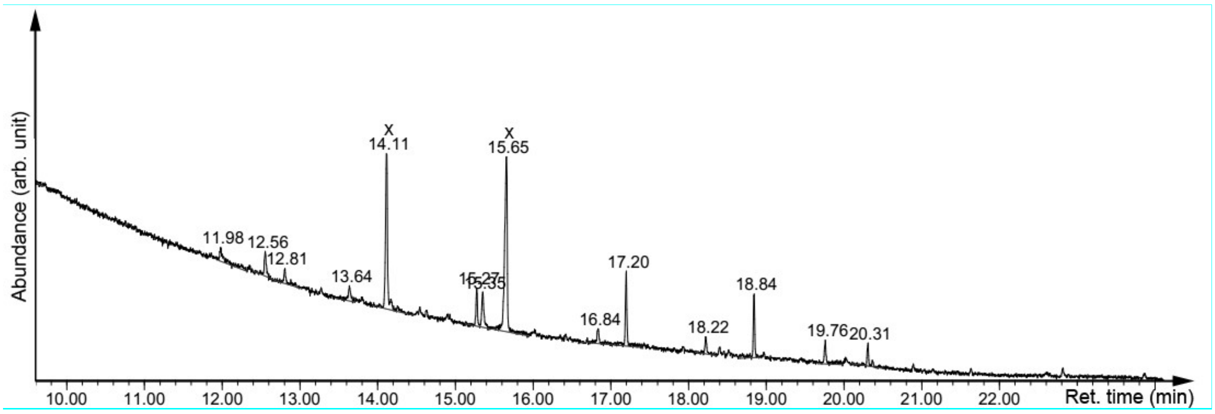

Supplementary Figure S8. GC-MS chromatogram of acid hydrolized/methylated products from *Rhodococcus degradans* BaTD-248 associated EPS. Peaks labeled by x belong to impurities.

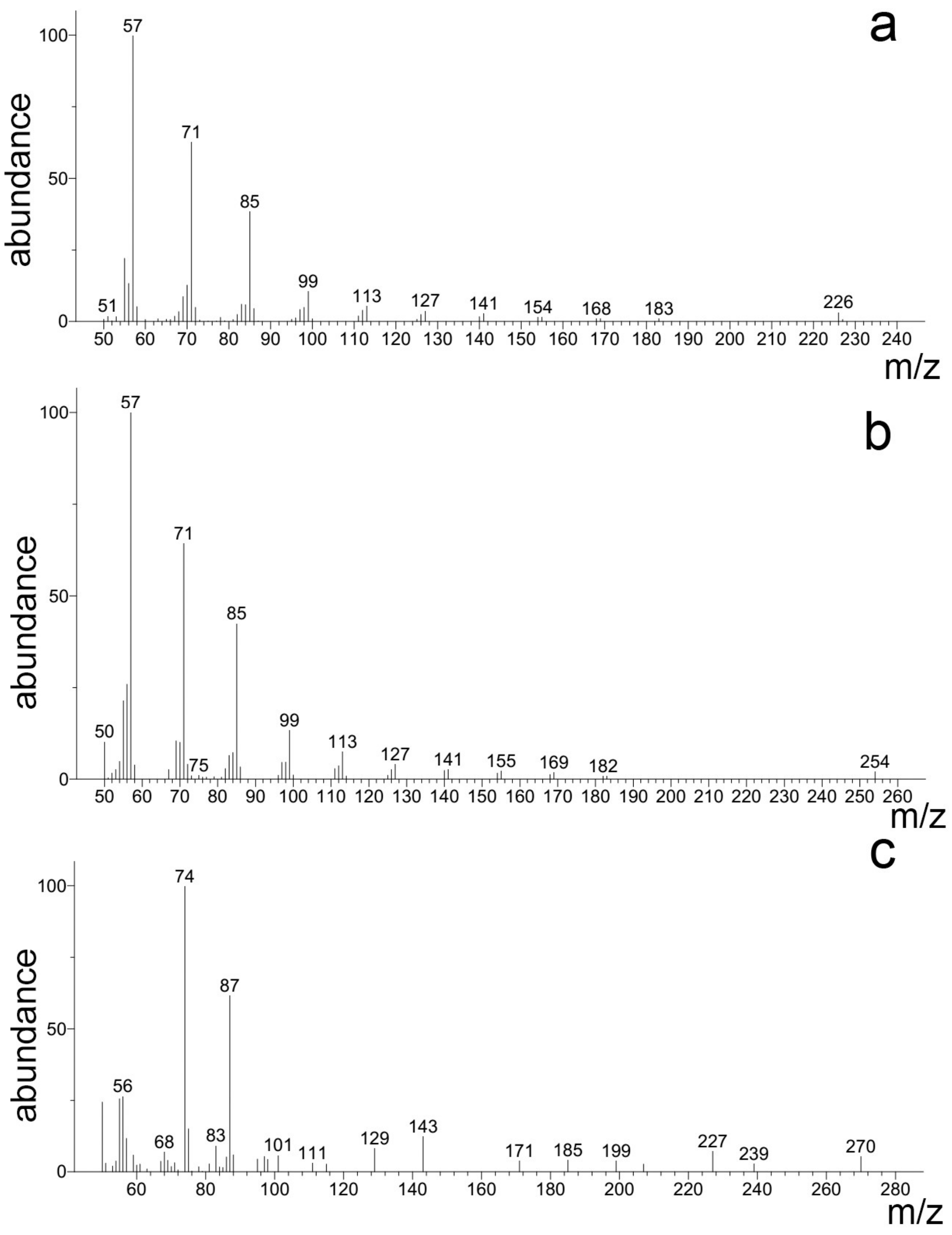

Supplementary Figure S9. Representative MS spectra of long-chain carbon components (ret. time **a**= 17.20 min, ret. time **b** = 18.84, ret. time **c**= 19.76) from the methylated mixture.

Supplementary Table S1. Phenotypic characteristics of the bacterial isolates. Strains: 1, *Stenotrophomonas maltophilia* BaSD-214; 2, *Bacillus simplex* BaSD-223; 3, *Rhodococcus degradans* BaTD-248. Characters are scored as: +, positive; -, negative reaction.

**Characteristic**	**1**	**2**	**3**
Cell size (µm)	1-2.5	2-4	1-2
Endospore	-	+	-
Colony colour	transparent	creamy white	pale pink
Mucoid colony	+	+	+
Hydrolysis of casein	+	+	-
Hydrolysis of gelatin	+	+	-
Hydrolysis of starch	-	-	-
Hydrolysis of Tween 80	+	-	-
Phosphatase	+	+	+
H_2S production from cysteine	+	+	-
NH_3 production from peptone	+	-	+
Hydrolysis of DNA	+	+	+
Aerobic Hugh-Leifson test	-	+	-
Anaerobic Hugh-Leifson test	-	-	-
Urease	+	+	+
Reduction of nitrate to nitrite	+	+	+
Reduction of nitrate to ammonia	+	-	-
Reduction of nitrate to nitrogen	-	-	-
Voges-Proskauer tests	-	-	-
API 50CH test:			
Glycerol	-	+	+
Erythritol	-	-	-
D-arabinose	-	-	-
L-arabinose	-	-	-
D-ribose	-	+	+
D-xylose	-	-	-
L-xylose	-	-	-
D-adonitol	-	-	-
Methyl-β-D-xylopyranoside	-	-	-
D-galactose	-	-	-
D-glucose	+	+	-
D-fructose	+	+	+
D-mannose	-	-	-
L-sorbose	-	-	-
L-rhamnose	-	+	-
Dulcitol	-	-	-
Inositol	-	-	+
D-mannitol	-	+	+
D-sorbitol	-	+	+
Methyl-α-D-mannopyranoside	-	-	-

Supplementary Table S1. continued

Methyl-α-D-glucopyranoside	-	-	-
N-acetyl-glucosamine	-	-	-
Amygdalin	+	+	-
Arbutin	-	+	-
Esculin	+	+	+
Salicin	+	+	-
D-cellobiose	-	+	-
D-maltose	+	+	-
D-lactose	-	-	-
D-melibiose	-	-	-
D-saccharose	-	+	-
D-trechalose	-	+	+
Inulin	-	-	-
D-melezitose	-	-	-
D-raffinose	-	-	-
Amidon	+	+	-
Glycogen	-	+	-
Xylitol	-	-	-
Gentiobiose	-	-	-
D-turanose	-	-	-
D-lyxose	-	-	-
D-tagatose	-	-	-
D-fucose	-	-	-
L-fucose	-	-	-
D-arabitol	-	-	+
L-arabitol	-	-	-
Potassium-gluconate	-	-	-
Potassium-2-ketogluconate	-	-	-
Potassium-5-ketogluconate	-	-	-

Supplementary Table S2. The main decomposition products released in the Py-GC/MS experiments from *Rhodococcus degradans* BaTD-248 associated EPS. Peak numbers refer to Fig. S4. Character is scored as: n. d. not determined.

Peak #	Ret. time (min)	Compound	m/z	M (g/mol)	Possible source
1	2.13-2.65	CO_2	28, 44	44	Each biopolymer
		Water	18, 17	18	Each biopolymer
		n-Pentene	42, 55, 70	70	Lipids
2	2.78	n-Hexane + 1-Hexene	43, 57 + 41, 56, 42	86 + 84	Lipids
3	3.53	n-Heptane + 1-Heptene	43, 57, 71 + 41, 56, 70	100 + 98	Lipids
4	4.84	Acetic acid	43, 45, 60	60	Lipids
5	5.29	n-Octane + 1-Octene	45, 57 + 43, 55, 70	114 + 112	Lipids
6	5.43	1-Hydroxy-2-propanone	43, 31, 74	74	Carbohydrates
7	5.64	Toluene	91, 92, 65	92	Protein
8	7.95	n-Nonane + 1-Nonene	45, 57 + 43, 55, 70	128 + 126	Lipids
9	10.81	n-Decane + 1-Decene	45, 57 + 43, 55, 70	142 + 140	Lipids
10	13.53	n-Undecane + 1-Undecene	43, 57, 71 + 41, 55, 70	156 + 154	Lipids
11	13.85	Undecene	55, 69, 154	154	Lipids
12	15.51	2-Hydroxy-2-methyl-2-cyclopentene-1-one	112, 55, 69	112	Carbohydrates
13	16.07	n-Dodecane + 1-Dodecene	57, 43, 71 + 55, 69, 83	170 + 168	Lipids
14	16.46	Phenol	94, 66, 65	94	Proteins
15	18.41	n-Tridecane	57, 43, 71	184	Lipids
16	18.44	1-Tridecene + 4-Methyl-phenol	55, 69, 83 + 107, 108, 77	182 + 108	Lipids, Proteins
17	18.97	Benzylnitrile	117, 90, 116	117	Proteins
18	20.62	n-Tetradecane	57, 43, 71	198	Lipids
19	20.67	1-Tetradecene	55, 69, 83	196	Lipids
20	21.17	3-Pyridinol	95, 69, 68	95	Proteins
21	22.71	n-Pentadecane	57, 43, 71	212	Lipids
22	22.76	1-Pentadecene	55, 69, 83	210	Lipids
23	23.50	1H-Indole	117, 90, 89	117	Proteins
24	24.68	n-Hexadecane	57, 71, 43	226	Lipids
25	25.03	3-Methyl-1H-indole	130, 131, 77	131	Proteins
26	26.32	Heptadecene	55, 69, 83	238	Lipids
27	27.31	Dodecanoic acid (Lauric acid)	73, 60, 129	200	Lipids
28	28.36	2-Pentadecanone	58, 71, 226	226	Lipids
29	29.19	Tetradecanitrile	43, 41, 97	208	Lipids
30	29.83	1,6-Anhydro-β-D-glucopyranose (Levoglucosan)	60, 57, 73	162	Carbohydrates
31	30.71	Tetradecanoic acid (Myristic acid)	73, 60, 129	228	Lipids
32	30.89	5-(2-Methylpropyl)-2,4-imidazolidinedione	100, 113, 57	156	Proteins

Supplementary Table S2. continued

33	31.77	2-Heptdecanone	58, 59, 43	254	Lipids
34	32.49	Thymine	126, 55, 54	126	DNA
35	32.61	Pentadecanenitrile	97, 57, 110	222	Lipids
36	33.84	Hexadecanoic acid (Palmitic acid)	73, 60, 129	256	Lipids
37	34.02	Fatty acid like compound	100, 113, 57	n.d.	Lipids
38	34.78	Glutamic acid derivative	84, 28, 41	n.d.	Protein
39	35.09	Tetradecanamide	59, 72, 86	227	Lipids
40	35.54	Octadecenenitrile (Oleanitrile)	55, 122, 136	263	Lipids
41	36.67	Fatty acid like compound	55, 69, 83, 264	n.d.	Lipids
42	38.01	Hexadecanamide (Palmitamide)	59, 72, 128	255	Lipids
43	40.53	Octadeceneamide (Oleamide)	59, 72, 55	281	Lipids
44	43.28	Proline-pyroglutamine	70, 208, 96, 124	208	Protein
